# Optimized Nutrition in Mitochondrial Disease Correlates to Improved Muscle Fatigue, Strength, and Quality of Life

**DOI:** 10.1007/s13311-023-01418-9

**Published:** 2023-09-18

**Authors:** Donna DiVito, Amanda Wellik, Jessica Burfield, James Peterson, Jean Flickinger, Alyssa Tindall, Kimberly Albanowski, Shailee Vishnubhatt, Laura MacMullen, Isaac Martin, Colleen Muraresku, Elizabeth McCormick, Ibrahim George-Sankoh, Shana McCormack, Amy Goldstein, Rebecca Ganetzky, Marc Yudkoff, Rui Xiao, Marni J. Falk, Maria R. Mascarenhas, Zarazuela Zolkipli-Cunningham

**Affiliations:** 1 Clinical Nutrition Department, Children’s Hospital of Philadelphia, Philadelphia, PA USA; 2 Department of Pediatrics, Division of Human Genetics, Mitochondrial Medicine Frontier Program, Children’s Hospital of Philadelphia, Philadelphia, PA USA; 3 Division of Gastroenterology and Nutrition, Children’s Hospital of Philadelphia, Philadelphia, PA USA; 4 Division of Endocrinology, Children’s Hospital of Philadelphia, Philadelphia, PA USA; 5 Department of Pediatrics, University of Pennsylvania Perelman School of Medicine, Philadelphia, PA USA; 6 Department of Biostatistics, Epidemiology and Informatics, University of Pennsylvania Perelman School of Medicine, Philadelphia, PA USA

**Keywords:** Primary mitochondrial disease, Nutritional intake, Macronutrients, Malnutrition, Inadequate calorie intake

## Abstract

We sought to prospectively characterize the nutritional status of adults ≥ 19 years (n = 22, 27% males) and children (n = 38, 61% male) with genetically-confirmed primary mitochondrial disease (PMD) to guide development of precision nutritional support strategies to be tested in future clinical trials. We excluded subjects who were exclusively tube-fed. Daily caloric requirements were estimated using World Health Organization (WHO) equations to predict resting energy expenditure (REE) multiplied by an activity factor (AF) based on individual activity levels. We developed a Mitochondrial Disease Activity Factors (MOTIVATOR) score to encompass the impact of muscle fatigue typical of PMD on physical activity levels. PMD cohort daily diet intake was estimated to be 1,143 ± 104.1 kcal in adults (mean ± SEM, 76.2% of WHO-MOTIVATOR predicted requirement), and 1,114 ± 62.3 kcal in children (86.4% predicted). A total of 11/22 (50%) adults and 18/38 (47.4%) children with PMD consumed ≤ 75% predicted daily Kcal needs. Malnutrition was identified in 16/60 (26.7%) PMD subjects. Increased protein and fat intake correlated with improved muscle strength in those with insufficient daily Kcal intake (≤ 75% predicted); higher protein and fat intake correlated with decreased muscle fatigue; and higher protein, fat, and carbohydrate intake correlated with improved quality of life (QoL). These data demonstrate the frequent occurrence of malnutrition in PMD and emphasize the critical need to devise nutritional interventions to optimize clinical outcomes.

**Supplementary Information:**

The online version contains supplementary material available at 10.1007/s13311-023-01418-9.

## Introduction

Primary mitochondrial disease (PMD) is a clinically heterogeneous group of > 300 distinct gene disorders that collectively affect at least 1 in 4,300 people across all ages [1]. Pathogenic mutations in either nuclear DNA or mitochondrial DNA (mtDNA) genes cause PMD. The clinical manifestations are multi-systemic and heterogeneous, resulting from impaired energy metabolism. The most common symptoms include muscle weakness, muscle fatigue, exercise intolerance, gastrointestinal symptoms, and imbalance [2]. No FDA-approved therapies exist for PMD [3].

Mitochondria provide cellular energy in the chemical form of adenosine triphosphate (ATP). Mitochondrial bioenergetics is based on the availability of reducing equivalents (NADH, FADH_2_), consumed as carbohydrate (CHO), protein (PRO), and fat, which react with oxygen (O_2_) via oxidative phosphorylation (OXPHOS) that takes place in the mitochondrial respiratory chain [4]. Mitochondrial function is determined by a tight coordination between mtDNA, nuclear DNA, and the metabolic state of the cells, which may be highly influenced by diet [5]. The association of nutrient oversupply, insulin resistance, and mitochondrial dysfunction has been extensively documented [6, 7]. Likewise, caloric restriction, as defined by a mild-to-moderate (20–40%) reduction in calorie intake compared with an ad libitum diet, has been shown to extend the lifespan of *C. elegans* [8], mice [9], rats [10], and monkeys [11], by acting on highly regulated pathways involving SIRT1 and PGC1α [12]. However, focused studies of dietary macronutrient composition and its effect on mitochondrial function have yielded conflicting results [13, 14]. A *Drosophila melanogaster* study showed that flies fed with a high protein:carbohydrate (P:C ratio) diet had higher citrate synthase activity [13], a marker of mitochondrial content. A low PRO diet containing 5% casein in rats was associated with induction of mitochondrial oxidative stress and impaired mitochondrial electron transport chain (ETC) enzyme activity [15]. Conversely, restricted intake of the essential amino acid methionine (0.17% methionine diet) in rats was associated with mitochondrial biogenesis in adipose tissue and increased mitochondrial aerobic capacity with decreased oxidative stress in liver and skeletal muscle [16, 17]. Thus, the effects of modified macronutrient intake on mitochondrial function requires further study.

Nevertheless, it is evident that mitochondria sit at the interface of nutritional caloric intake and organ energy requirements [12]. Nutritional modulation holds considerable promise as a therapeutic intervention in PMD [18], although we lack understanding of the precise modulatory approach to be implemented. The effect of dietary nutraceuticals implemented as standard of care in PMD [19] mimic the nutritional benefits of diet, which include antioxidant effects, yielding electron donors or cofactors in the mitochondrial ETC, and augmenting ATP production [20]. The ketogenic diet has had reported success in some cases of PMD-related seizures [21, 22], but is not considered standard of care in PMD due to insufficient evidence of its efficacy and safety, except in patients with pyruvate dehydrogenase complex (PDCD) deficiency [23]. Further, PMD patients express symptoms that may impair nutritional intake, such as dysphagia, GI dysmotility, and anorexia [24]. Few studies have been conducted to report nutritional status in PMD patients. Zweers et al. reported inadequate caloric intake in an adult PMD cohort (n = 80) [25] and showed that PRO intake was moderately correlated to improved handgrip strength, with a high prevalence of malnutrition (n = 27/37 adult subjects, 73%) [26], concluding that nutritional assessments is warranted in PMD.

**Fig. 1:**
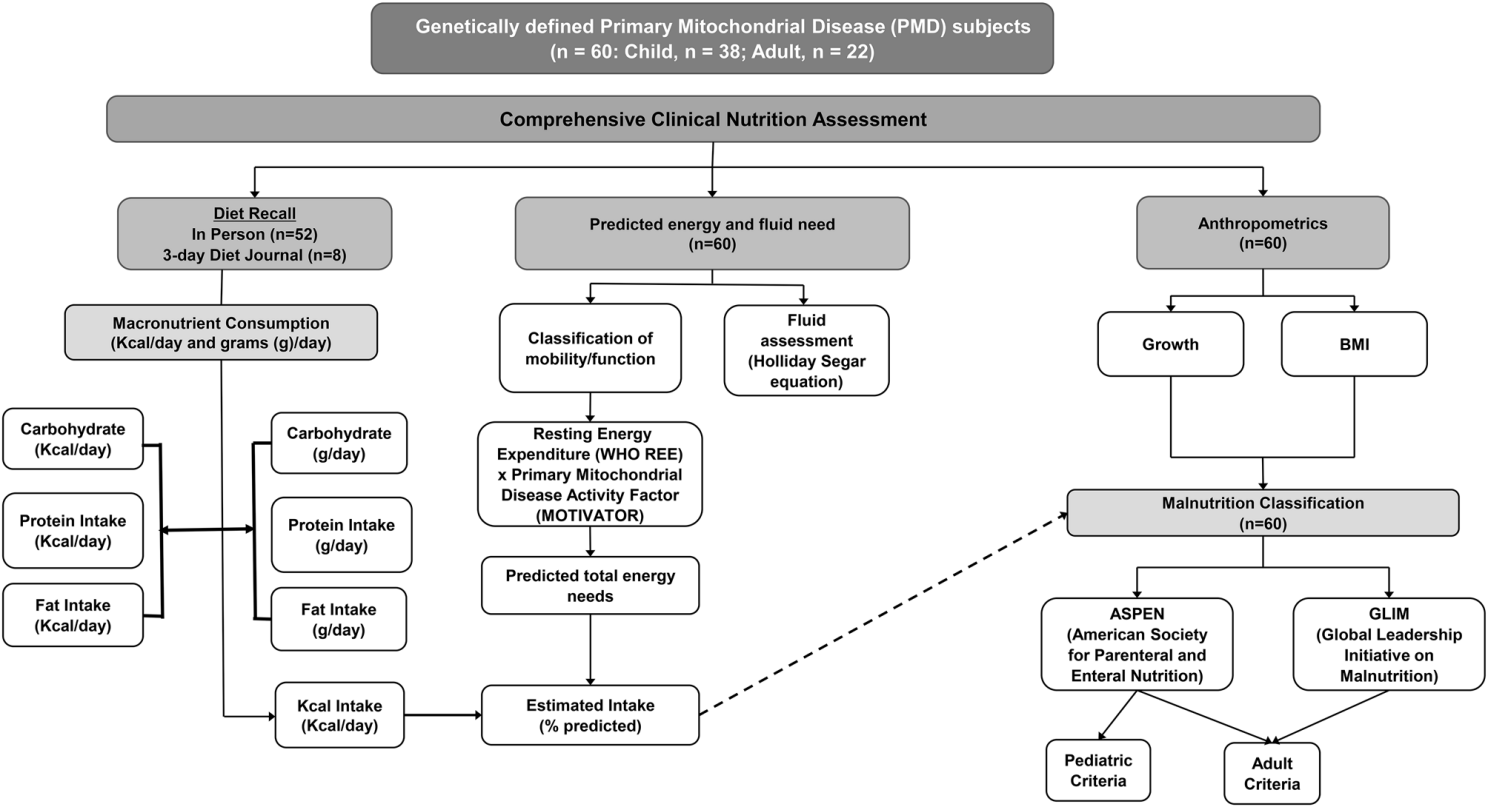
**Comprehensive nutritional evaluation in primary mitochondrial disease**. Figure 1 displays the main study outcomes evaluated in a PMD cohort (n = 60) consisting of child (n = 38) and adult (n = 22) subjects. A comprehensive nutritional assessment was performed to evaluate the predicted and estimated daily Kcal intake, macronutrient consumption (Kcal/day and grams (g)/day), growth, and assessment of malnutrition in all subjects

In this single-center prospective study, we sought to precisely characterize the baseline nutritional status [27] of our genetically-confirmed PMD adult and child patient cohort (n = 60, Fig. 1 study overview). We further assessed the relationship of daily Kcal and macronutrient intake to validated myopathy outcome measures [28] and quality of life (QoL) scores, to guide the development of nutritional management strategies that may be tested in future clinical trials in PMD.

## Methods

### Statistics

Cohort demographics were summarized by standard descriptive statistics. Nutritional results are expressed as percent predicted. Group comparisons were performed using two-sample t-test or ANOVA or their non-parametric equivalent for continuous variables, and Chi-squared or Fisher’s exact test for categorical variables, as appropriate. Pearson’s or Spearman’s correlations as appropriate were used to assess relationships between nutritional results and objective measures and survey results, respectively. Analyses were conducted in Prism (Version 8.3, San Diego) and RStudio [29]. The Zanthro function (Stata 17.0) was used to calculate z-scores: CDC growth charts were used as reference data for weight-for-age, height-for-age, and BMI z-scores for children > 2 years of age, and WHO growth charts for children ≤ 2 years of age.

### Study Design and Approval

Figure 1 represents an overview of this prospective, observational study. We conducted full nutritional assessments (Supplemental Fig. 1) in 22 adults and 38 children with genetically diagnosed PMD. We excluded subjects (2 adults and 6 children; Table 1) who were exclusively fed via gastric (or gastroduodenal/gastrojejunal) tube, but we included those who received G-tube feeds as a supplement to oral intake. All subjects were prescribed mitochondrial supplements as standard of care [30]. Subjects baseline nutritional evaluations were conducted in the Children’s Hospital of Philadelphia (CHOP) Mitochondrial Medicine Frontier Program (MMFP) between May 2018 and February 2020. All subjects were consented to CHOP IRB protocols #6177 (MJF, PI) or #013364 (ZZC, PI).

**Table 1:** Subject demographics

**Parameter (mean ± SD)**	**Adults** ≥ 19 years **(n = 22)**	**Pediatric** **(n = 38)**	
Age (years, range)^a^	35.0 ± 13.0 (20.2 – 64.5)	9.5 ± 5.1 (0.6 – 18.2)	
Male gender, number (%)	6 (27%)	23 (61%)	
Weight (kg, range)	58.7 ± 13.0 (35.1 – 88.5)	29.0 ± 17.8 (6.1 – 92.9)	
Weight for age z-score (mean z-score ± SD, range)^b^	NA	-1.7 ± 2.9 (-13.6 – 2.1)	
Height (m, range)	1.63 ± 0.1 (1.4 – 1.8)	1.26 ± 0.29 (0.7 – 1.0)	
Height for age z-score (mean z-score ± SD, range)^b^	NA	-1.1 ± 1.8 (-7.3 – 2.5)	
BMI (mean ± SEM)	23.4 ± 1.4	16.7 ± 0.7	
BMI z-score (Mean z-score ± SD, range)^b^	NA	-1.04 ± 0.3 (-6.2 – 2.0)	
G-tube in-situ, number (%)	2 (9.1%)	6 (15.8%)	
Laboratory Marker (mean ± SEM)	**Adults** ≥ 19 years **(n = 22)**	**Pediatric** **(n = 38)**	**T-test p-value**
Plasma Creatine Kinase (U/L)	406.9 ± 202.7 (30–135) n = 16	131.0 ± 22.55 (60–335) n = 27	0.10
Creatinine (mg/dL)	0.7 ± 1.3 (0.1–0.75) n = 16	0.6 ± 0.05 (0.4–1.3) n = 23	0.003
Albumin (g/dL)	4.5 ± 0.07 (3.5–5.0) n = 18	4.5 ± 0.06 (3.7–5.6) n = 32	0.80
Cholesterol (mg/dL)	169.7 ± 13.73 (107–217) n = 10	159.4 ± 7.01 (126–231) n = 14	0.63
Plasma Triglycerides (mg/dL)	149.2 ± 29.53 (37–140) n = 10	110.0 ± 13.47 (34–165) n = 15	0.44
Blood Pyruvate (mM)	0.14 ± 0.02 (0.05–0.14) n = 17	0.16 ± 0.04 (0.05–0.14) n = 25	0.47
Blood Lactate (mM)	2.6 ± 0.63 (0.91–11.19) n = 18	2.9 ± 0.48 (0.85–13.0) n = 27	0.36

^a^Age of adult ≥ 19 years

^b^BMI, weight for age, and height for age z-scores were only calculated for pediatric subjects

### Nutritional Assessment

The registered dietitian nutritionist (RDN) conducted standardized diet interviews of nutritional intake within the past month at the clinic visit. A small subset of subjects (2 adults, 6 children) completed 3-day food records prior to the visit. The RDN entered all data into a nutrient analysis program, MetabolicPro (Genetic Metabolic Dieticians International, GDMI), which analyzed daily Kcal and macronutrient intake. Macronutrient intake (grams (g)/day) was compared to the Dietary Reference Intakes (DRI) [31] for CHO and PRO, and to a population study published in the DRI [31] for fat; while the distribution of dietary macronutrients (Kcal/day) was compared to the Recommended Daily Allowance (RDA) for age [32]; as well as the acceptable macronutrient distribution range (mid-point AMDR) [31]. Mean daily fluid intake was compared to predicted fluid intake using the Holliday Segar method [33]. Energy needs were calculated using the World Health Organization (WHO) resting energy expenditure (REE) equation [32] multiplied initially by an ASPEN/RDA activity factor (AF) based on physical activity levels [32, 55, 56].

### Development of PMD-Specific AFs

We reviewed existing literature on AFs and gross motor function classifications [32, 34–36, 55] in our rigorous approach to develop the Mitochondrial Activity Factors (MOTIVATOR score). On close review, it became apparent that ASPEN/RDA AFs use muscle strength as the main indicator of physical activity, whereby fatigue and exercise intolerance, which are also determinants of mobility and commonly observed in PMD [2], are not considered. We utilized our clinical expertise in PMD phenotype (DD, JF, ZZC) as characterized by our Mitochondrial Myopathy Composite Assessment Tool (MM-COAST) assessments of myopathy [28] to establish appropriate ranges of MOTIVATOR AF’s corresponding to the individual’s gross motor and mobility status, taking into account the presence or absence of fatigue and exercise intolerance that is typical of MM [28]. It is standard practice for RDNs to work within AF ranges. In this study, the lower limits of the MOTIVATOR-AF range (Tables e-6–8) were applied in accordance with standard RDN practice. A decision to apply the upper limit AF at each individual activity level should be based on clinical judgement. We incorporated overlap in AFs between consecutive mobility levels, eg. the upper limit of the AF range in the ‘very light’ and the lower limit in the ‘light’ activity levels are both at 1.3 in the Pediatric MOTIVATOR activity level (Table e-7). PMD activity levels are known to fluctuate over the course of a day, or across several days, related to varying prominence of their symptoms of fatigue and exercise intolerance, which results in variability in motor function capabilities. Further, the RDN can apply modifications to the AF based upon increased (add a range of 0.1- 0.3) or decreased (subtract a range of 0.1- 0.3) energy demands, or fatigue (score down one activity level and apply the highest AF from the stated range of that level) if an individual requires 1–2 days of rest after participating at a specific activity level (Tables e-6–8). Thus, the assigned MOTIVATOR AFs are individualized and appropriately capture the impact of fatigue. MOTIVATOR AF levels were correlated to the MM-Composite Assessment Tool (MM-COAST) Composite Score of myopathy [28] and the 6-minute walk test (6MWT) measure of exercise intolerance and fatigue.

### Malnutrition Assessment

Adult subjects were assessed for malnutrition using the Academy of Nutrition and Dietetics/American Society for Parenteral and Enteral Nutrition (AND/ASPEN) criteria [37]. Malnutrition is defined as deficiencies, excesses, or imbalances in a person’s intake of energy and/or nutrients and includes those in the groups of undernutrition, micronutrient-related malnutrition, and overweight/obesity [38]. Of the six criteria, the two specific indicators used in this study included (i) reduced Kcal intake of < 75% predicted for > 30 days and (ii) percent (%) weight loss of 5% within 1 month, 7.5% in 3 months, 10% in 6 months, or 20% (> 12 months). Malnutrition assessment was then confirmed against the European Global Leadership Initiative on Malnutrition (GLIM) [39]. In the pediatric subjects, the Pediatric ASPEN criteria [40] were utilized.

### Anthropometrics Measurement

Height and weight measurements were performed by the clinic medical assistants (MAs) trained in standardized approach and re-measured by the RDN when needed. If standing was not possible, weight was subtracted from the combined weight with a caregiver/wheelchair (Tronix scales). Height measurements were obtained in standing or recumbent positions (Seca stadiometer), or via arm span by the RDN. BMI classification followed standard classifications in adults and children [41, 55]. If a subject was overweight or obese, an adjusted body weight was calculated [42].

### Myopathy Objective Assessments

PMD subjects (n = 33/60, 55%) completed objective assessments including hand-held dynamometry for muscle strength [43–46], 30 second Sit-to-Stand test (30s STS) [47], 6-minute walk test (6MWT) [48], and elbow dynamometry repetitions for muscle fatigue. A Mitochondrial Myopathy Composite Assessment Tool (MM-COAST) Composite Score that is reflective of all objective measures was calculated [28]. PMD subjects (n = 37/60, 61.7%) completed a series of surveys: the Pediatric Quality of Life Inventory (PedsQL) [49, 50]; Modified Fatigue Impact Scale (MFIS) [51]; the (Kanofsky/Lansky (K/L) Performance Scale [52]; and the Pediatric Evaluation of Disability Inventory-Computer Adaptive Test (PEDI-CAT) [53]. All data were collected in REDCap (Version 9.10), and presented as mean z-score ± standard deviation [35] for PedsQL and PEDI-CAT, and as mean score ± standard error mean (SEM) for MFIS and K/L.

## Results

### A. General Results

#### Demographics

A total of 60 adult and pediatric subjects with genetically-confirmed PMD were studied. 38/60 (63.3%) were children ≤ 18 years of age, which included 2 infants (< 12 months), 15 children (1-9 years), and 21 adolescents (10–18 years) (Table 1).

Genetic etiologies were identified in mtDNA in 35 subjects (58.3%), and in nuclear DNA in 25 subjects (41.7%, Table e-1). The most frequent genetic etiology was single large-scale mtDNA deletions (SLSMD, 10/60, 16.7%). Eight subjects (13.3%) had a gastrostomy (G)-tube in place for supplemental feeds, including 2 adults and 6 children (Table 1). The two adults who presented with a G-tube, respectively, had SLSMD (n = 1) and a nuclear gene variant in *TWNK* (c.1110C > G: p.F370L) (n = 1). In the 6 pediatric subjects with G-tubes, 4/6 (66.7%) had mtDNA-disease etiologies including SLSMD (n = 2), *MT-ND5* (n = 1), and *MT-ND3* (n = 1), while 2/6 carried nuclear genetic etiologies (*SURF1* (n = 1) and *POLG* (n = 1)).

#### Body Mass Index (BMI) & Weight Classification

**Fig. 2:**
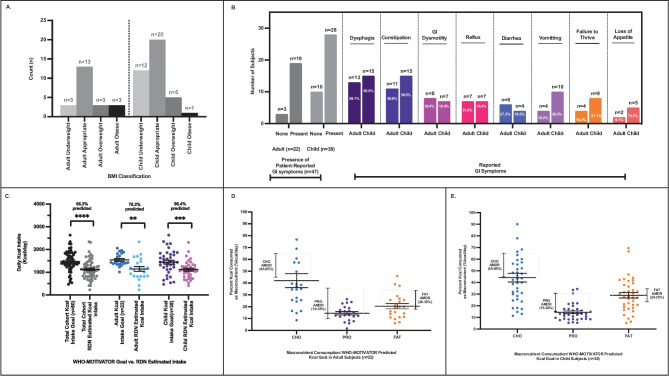
**A** BMI classification. BMI of subjects based on ASPEN criteria for adult and child subjects is displayed. Most subjects had appropriate BMI. 3/22 (13.6%) adults and 12/38 (31.6%) children were underweight. **B** Patient-reported gastrointestinal symptoms (GI). 47/60 (78.3%) PMD subjects reported at least one GI symptom. The most commonly reported GI symptoms were dysphagia (28/60, 46.7%), constipation (26/60,43.3%), GI dysmotility (15/60, 25%), and reflux (14/60, 23.3%). **C** Predicted and dietician (RDN)-estimated daily Kcal intake in adult and child subjects. The predicted Kcal goal (WHO Resting Energy Expenditure value x MOTIVATOR Activity Factor) is compared to the RDN-estimated Kcal intake in all subjects (n = 60) and stratified by age. Results show the significantly lower RDN-estimated cohort mean daily Kcal intake of 1,125 ± 54.4 kcal/day to the predicted Kcal intake values (86.3% predicted, n = 60, *p* < 0.0001****). In the adult subjects, the estimated Kcal intake was 1,143 ± 104.1 kcal/day (76.2% predicted, n = 22, p = 0.003**), while in the child subjects, the estimated Kcal intake was 1,114 ± 62.3 kcal/day (86.4% predicted, n = 38, p = 0.001***). **D** Macronutrient consumption/WHO-MOTIVATOR predicted Kcal goal in adult PMD subjects (%Kcal/day) (n = 22). In adult subjects, mean macronutrient consumption percentage of WHO-MOTIVATOR predicted Kcal intake for CHO was 42.1 ± 5.9% Kcal (76.5% predicted, 627.6 ± 81 kcal/day), 14.5 ± 1.4% Kcal (96.8% predicted, 219.2 ± 18.5 kcal/day) for PRO, and 20.5 ± 2.3% Kcal (68.2% predicted, 306.1 ± 29.6 kcal/day) for fat. **E** Macronutrient consumption/WHO-MOTIVATOR predicted Kcal goal in child PMD subjects (%Kcal/day) (n = 38). In child subjects, mean macronutrient consumption percentage of WHO-MOTIVATOR predicted Kcal intake for CHO was 44.1 ± 3.8% Kcal (89.7% predicted, 571.1 ± 40.7 kcal/day), 14.3 ± 1.2% Kcal (71.7% predicted, 190.0 ± 12.8 kcal/day) for PRO, and 29.0 ± 2.3% Kcal (94.2% predicted, 365.1 ± 23.4 kcal/day) for fat

Mean weight and height for adults (n = 22) was 58.7 ± 13.0 kg and 1.63 ± 0.1 m, respectively, and 29.0 ± 17.8 kg (mean z-score -1.7 ± 2.9; range -13.6 to 2.1) and 1.26 ± 0.29 m (mean z-score -1.1 ± 1.8; range -7.3 to 2.5) in the child subjects (n = 38) (Table 1). Mean BMI was 23.4 ± 1.4 in adults, and 16.7 ± 0.7 (mean z-score -1.04 ± 0.3; range -6.2 to 2.0) in child subjects. Weight classification was determined using BMI classification in adults and BMI percentile (%) in children [55, 56]. Of the 22 adults, 3/22 (13.6%) adults were underweight (BMI ≤ 18.5 kg/m^2^), 13/22 (59.1%) had appropriate weight (BMI 18.5 – 25 kg/m^2^), and 6/22 (27.3%) were overweight/obese (BMI ≥ 25 kg/m^2^). Of the 38 children, 12/38 (31.6%) were underweight (BMI percentile < 5th), 20/38 (52.6%) were appropriate (BMI percentile 5-84th), and 6/38 (15.8%) were overweight/obese (BMI percentile > 85th) (Table e-2, Fig. 2A). Of the six pediatric subjects with a G-tube, 4/6 (66.7%) were underweight, while the remaining were appropriate weight or overweight, 1/6 (16.7%) each.

#### Prevalence of Gastrointestinal (GI) Symptoms

Patient and/or physician-reported GI symptoms were extracted from the electronic medical record (EMR) primary care physician and/or GI physician clinical evaluation letter. At least one GI symptom was reported in 28/38 (73.7%) of child subjects and 19/22 (86.4%) adults. Across the PMD cohort (n = 60), the most frequent GI symptoms were dysphagia (Human Phenotype Ontology, HPO ID 0002015) (28/60, 46.7%) and constipation (HPO ID 0002019) (26/60, 43.3%). Of those subjects with constipation, 10/26 required treatment for constipation, 10/26 were managed with diet, and 6/26 did not require treatment. In adults subjects, dysphagia was the most frequent GI symptom (13/22, 59.1%), while both dysphagia and constipation were most frequent in children (15/38, 39.5%). GI dysmotility (HPO ID 0002579) (15/60, 25%), vomiting (HPO ID 0002013) (14/60, 23.3%), gastroesophageal reflux (HPO ID 0002020) (14/60, 23.3%), failure to thrive (HPO ID 0001508) (12/60, 20%), and diarrhea (HPO ID 0002014) 10/60 (16.7%) were also reported. Nausea (HPO ID 0002018), abdominal pain (HPO ID 0002027), loss of appetite (HPO ID 0004396), poor weight gain (HPO ID0001508), and feeding difficulties (HPO 0011968) were each reported in < 15% of the cohort (Table e-3, Fig. 2B).

Among those who consumed 75.01–100% of their predicted daily Kcal needs (n = 14/60, 23.3% of the total cohort), 4/14 (28.6%) reported no GI symptoms, 1/14 (7.14%) reported 1 symptom, and 9/14 (64.3%) subjects reported having between 2 to 7 GI symptoms. The most common symptoms were dysphagia in 6/14 (42.9%) and constipation in 5/14 (35.7%). Of those who consumed ≤ 75% predicted Kcal/day (n = 29/60, 48.3% of the total cohort), which is consistent with malnutrition consumption levels [37, 40], 5/29 (17.2%) reported 1 or less GI symptoms, and 24/29 (82.8%) reported up to 7 GI symptoms. Constipation in 15/29 (51.7%) and dysphagia in 12/29 (41.4%) were the most common symptoms. Fisher’s exact test revealed no significant difference in the frequency of GI symptoms between the ≤ 75% and 75.01–100% Kcal intake groups, p = 0.08.

Of the underweight subjects based on BMI (n = 15/60, 25%), 12/15 subjects (80%) reported up to 6 GI symptoms, while 3/15 (20%) subjects reported no symptoms. The most common symptom was dysphagia in 10/12 (83.3%) subjects. There were 5/15 (33.3%) underweight subjects who consumed ≤ 75% predicted Kcal/day. Among this subset, 2/5 (40.0%) reported 2–6 GI symptoms.

#### Laboratory Markers

Biochemical measurements performed in adult and child PMD obtained ≤ 6 months before or immediately after the baseline nutrition evaluation included plasma CK, creatinine, albumin, cholesterol, triglycerides, pyruvate, and lactate (Table 1, mean ± standard error of mean (SEM). Of note, mean blood lactate was elevated at 2.8 ± 0.38 (normal range 0.8-2 mM) in 45/60 subjects. Blood lactate levels were significantly different in subjects with malnutrition, (5.0 ± 1.0, range 0.97–12.03 mM, n = 13/45), compared to those subjects without malnutrition, (1.9 ± 0.16, range 0.85–5.13 mM, n = 32/45), p = 0.0002. Plasma amino acid analysis performed in 25/60 subjects revealed that plasma alanine trended higher in subjects with malnutrition, 589.8 ± 93.2 μmol/g, n = 4, compared to those without malnutrition, 421.8 ± 27.2 μmol/g, n = 21, p = 0.1. Increased plasma alanine levels are indicative of chronic lactic acidosis or gluconeogenesis [54]. There was no significant difference in plasma CK, albumin, cholesterol, creatinine, or triglycerides levels in subjects with malnutrition (n = 16/60) and without malnutrition (n = 44/60). Plasma GDF15 levels were elevated at 1,567 ± 258.7 (mean ± SEM, range 183–6,000, normal ≤ 750 pg/ml) across the PMD cohort (n = 59), specifically 1,882 ± 425.6 in adult (n = 17/59) and 1,283 ± 312.2 in child PMD subjects (n = 32/59). GDF15 levels did not correlate to weight (r = 0.26, p = 0.07) or height (r = 0.25, p = 0.09) measurements. Linear regression analysis of GDF15 levels to BMI was not significant, R^2^ = 0.01, p = 0.61. There was no significant difference in GDF15 levels in subjects with malnutrition (2,338 ± 822.2 pg/ml), n = 10, and without malnutrition (1,274 ± 232.5 pg/ml), n = 44, p = 0.61.

### B. Nutritional Evaluation Results

#### Estimation of Total Energy Expenditure in PMD Subjects Using the WHO Predictive Equation and ASPEN/RDA Activity Factors (AF)

Eight three-day diet records and 52 diet interviews were obtained on 60 patients followed at the Children’s Hospital of Philadelphia Mitochondrial Medicine Frontier Program. Resting energy expenditure (REE) was estimated using the World Health Organization (WHO) REE equation [32] multiplied by an ASPEN/RDA activity factor (AF) based on physical activity levels [32]. Predicted total energy expenditure (TEE) is the product of REE x AF [55, 56].

As our center evaluates the full age spectrum of PMD patients, our objective was to apply the same predictive REE equation across the ages in this study. The WHO equation is routinely utilized in children, but in adults (≥ 19 years), the Harris-Benedict (HB) [36] and Mifflin St. Jeor (MSJ) [57] equations are commonly used. We compared WHO-calculated REE to Harris-Benedict and Mifflin St. Jeor-REE estimates in the adult subjects to show that WHO-REE can be utilized in our adult PMD subjects. In conjunction with the ASPEN/RDA AFs, the predicted TEE was not significantly different between the three prediction equations, at 1,936 ± 59.0 kcal by WHO-REE x AF, 1,938 ± 50.1 kcal by HB-REE x AF, and 1,827 ± 62.5 kcal by MSJ-REE x AF, p = 0.30 (ANOVA, Table e-4). These data confirm that the WHO-REE equation can be appropriately administered to predict TEE across the PMD age spectrum.

We initially applied the ASPEN/RDA AFs in the prediction of daily Kcal intake to our cohort (Table e-5). RDN estimated dietary intake revealed a mean Kcal intake (mean ± SEM) of 1,143 ± 104.1 kcal/day in adults (range 226 – 2,340 kcal/day, n = 22), which is 60.1 percent (%) predicted (RDN estimated Kcal intake/WHO-REE x ASPEN/RDA AF predicted Kcal intake) (Table e-5, Fig. 2C). Mean RDN estimated Kcal intake in child subjects was 1,114 ± 62.3 kcal/day (range 410—2,314 kcal/day, n = 38) or 76.5% of that predicted by the WHO-REE x ASPEN/RDA AFs (Table e-5). Our data reveals that adult and child PMD subjects consumed significantly decreased daily Kcal intake (*p* < 0.0001) at baseline nutritional assessment prior to dietary intervention.

#### Development of PMD-Specific Activity Factors (MOTIVATOR) in the Estimation of Total Energy Expenditure (TEE)

Currently, guidance on AF selection in PMD does not exist. ASPEN/RDA AFs, which reflect the physical capacity of healthy individuals of average body composition [32, 55, 56], uses muscle strength as the main indicator of physical activity. Similarly, guidance on estimation of AFs in cerebral palsy (CP) [65] is based on muscle strength, as muscle weakness and/or spasticity is the main phenotype in CP. By contrast, there is a high prevalence of muscle fatigue and exercise intolerance with or without muscle weakness in PMD [2] that significantly impacts activity levels and thus energy expenditure. Individuals with PMD likely have reduced muscle mass [58], which leads to decreased energy needs. In addition, prevalence of low muscle tone and involuntary movements in PMD [59] should also be considered for estimation of energy expenditure.

To ensure a standardized approach to assigning AFs in predicting PMD energy needs, we established and propose the broad implementation of customized PMD-specific AFs, Mitochondrial Activity Factors (MOTIVATOR, Tables e-6–8). With the benefit of extensive clinical experience in characterizing PMD phenotype using a validated objective assessment of Mitochondrial Myopathy (MM), the MM-Composite Assessment Tool (MM-COAST) [28], and review of existing literature on AFs and gross motor function classifications [32, 34–36, 55], we defined clinically relevant ranges of AFs for child (Table e-6–7) and adult (Table e-6, e-8) PMD subjects that correspond to their motor function and fatigue levels, in order to facilitate a more personalized and rigorous estimation of TEE. In this study, the lower limit of the MOTIVATOR-AF range (Tables e-6–8) was consistently applied unless the upper limit was deemed more suitable based on clinical assessment. Routine RDN practice to consider growth patterns, muscle mass, acute illness, medications, involuntary movements [59], and mechanical ventilation (Table e-6) to estimate the AF should proceed as usual and be added to the selected MOTIVATOR-AF (Tables e-7–8). Determination of MOTIVATOR-AFs should be based on clinician assessments of mobility and fatigue levels and not by PMD subject self-report, which may be less accurate.

#### Estimation of TEE Using PMD-Specific Activity Factors (MOTIVATOR)

We anticipated that ASPEN/RDA AFs [31] would overestimate TEE as it overlooks the impact of exercise intolerance and fatigue on PMD mobility. In adult subjects, predicted TEE (WHO-REE x ASPEN/RDA AFs) was 1,936 ± 59.0 kcal/day, compared to 1,554 ± 58.0 kcal/day with WHO-REE x MOTIVATOR AFs, *p* < 0.0001. Thus, RDN estimated dietary intake in PMD subjects as percent (%) predicted of the TEE was 60.1% using ASPEN/RDA AFs compared to 76.2% with MOTIVATOR AFs, *p* < 0.0001 (t-test, Table e-5, Fig. 2C). In child subjects, predicted TEE was 1,663 ± 94.5 kcal/day (76.5% predicted dietary intake) with ASPEN/RDA AFs compared to 1,444 ± 84.9 kcal/day (86.4% predicted) with MOTIVATOR AFs, *p* < 0.0001 (t-test, Table e-5, Fig. 2C).

These data confirm that the MOTIVATOR predicts lower energy needs in PMD, which we consider to be more precise since MOTIVATOR-AFs were designed for the PMD phenotype, compared to ASPEN/RDA AFs that do not discriminate the various mobility levels of a PMD subject. Indeed, ASPEN/RDA AFs overestimated Kcal goals by 382 kcal (19.7%) in adults and 189 kcal (11.6%) in child subjects (Table e-5). Subsequent results presented in this manuscript are based on WHO-MOTIVATOR predicted total Kcal requirements.

#### Daily Macronutrient Consumption

##### a. Altered Macronutrient Consumption in PMD

Having confirmed inadequate daily Kcal intake across the PMD cohort, we sought to characterize their daily macronutrient distribution. Macronutrient consumption expressed as a fraction (percentage, %) of daily Kcal intake revealed mean carbohydrate (CHO) consumption to be 52.9% (96.2% predicted when compared to the Recommended Daily Allowances (RDA) [32], 20.2% protein (PRO) (134.7% predicted), and 27.4% fat (91.5% predicted) in adult PMD subjects (Table e-9). In child subjects, mean CHO calorie consumption was 50.1% (101.3% predicted), 17.5% PRO (87.5% predicted), and 33.5% fat (109.7% predicted) (Table e-9). Of note, 17.5% PRO calorie consumption in the child subjects remains within the acceptable macronutrient distribution range (AMDR) range of 15–30% [31]. These results reveal an apparently normal distribution of macronutrient consumption. However, total Kcal intake is essentially a measure of total macronutrient intake. Therefore, in the context of significantly low daily Kcal intake in our PMD cohort in whom overall nutritional needs are not being met, the macronutrient distribution would be expected to be deficient.

The adult RDA macronutrient distribution is based on a healthy adult ~ 2,000 kcal diet [32], of CHO 1,110 kcal/day (55%), PRO 300 kcal/day (15%), and fat 600 kcal/day (30%). Our adult PMD cohort mean consumption of each individual macronutrient was distinctly below these stated levels at 627.6 ± 81.0 kcal CHO, 219.2 ± 18.5 kcal PRO, and 306.1 ± 29.6 kcal fat (Table e-9), with an overall daily Kcal intake at 76.2% predicted. Thus, it is apparent that analysis of macronutrient distribution as a fraction of daily Kcal consumption is less informative when the estimated daily Kcal consumption is decreased, as was observed in our PMD cohort. We propose that when the estimated daily Kcal consumption is low, macronutrient distribution analysis would be more meaningful if interpreted in the context of the predicted amount of Kcal that should be consumed.

Indeed, when the macronutrient distribution was expressed as a fraction of predicted Kcal intake goals (WHO-REE x MOTIVATOR AFs), at a goal of 1,554 ± 58 kcal for adults (n = 22) and 1,444 ± 84.9 in children (n = 38) subjects, macronutrient distribution in adult subjects was 42.1% CHO calorie consumption (76.5% predicted in comparison to the RDA goal of 55% [32], [AMDR of 45–65%], 14.5% PRO (RDA goal 15%, [AMDR of 10–35%], 96.8% predicted), and 20.5% fat (RDA goal 30%, [AMDR 20–35%], 68.2% predicted). In child subjects, the macronutrient distribution was 44.1% CHO calorie consumption (RDA goal 50%, [AMDR 45–65%], 89.7% predicted), 14.3% PRO (20%, [15–30%], 71.7% predicted), and 29.0% fat (30%, fat [25–35%], 94.2% predicted) (Table e-9, Fig. 2D–E). These results indicate altered distribution of macronutrient consumption, in alignment with the inadequate daily Kcal intake in PMD subjects (Fig. 2D–E). Thus, macronutrient consumption should be routinely evaluated in PMD, in the context of estimated as well as predicted calorie goals, particularly if the estimated Kcal intake is inadequate. Subsequent analyses reported here express macronutrient consumption as a fraction of WHO-MOTIVATOR predicted Kcal intake goals.

##### b. Significantly Decreased Fat Intake in PMD Subjects

We proceeded to further characterize PMD macronutrient distribution by defining absolute intake in grams/day (g/day). We compared estimated CHO and PRO intake to DRI goals [32], and fat intake to population data published in the DRI [60], and reviewed in the context of recent National Health and Nutrition Examination Survey (NHANES) references (2017–2018) [61]. PMD adults were found to consume a mean of 156.9 ± 20.2 g (g)/day of CHO (DRI goal 130 g, 120.7% predicted), 54.8 ± 4.6 g/day of PRO (DRI goal 56 g/day for males, 46 g/day for females, 111.9% predicted), and 34.01 ± 3.3 g/day of fat (population mean [60] 75.4 ± 3.3 g/day, 47.2% predicted, Table e-10). This confirms that adult PMD fat intake was significantly decreased, *p* < 0.0001. In the child cohort, mean CHO intake was 142.8 ± 10.2 g/day (DRI goal 130 g/day, 111.2% predicted), 47.5 ± 3.4 g of PRO (age-dependent DRI, (Table e-10), overall 186.1% predicted), and 40.6 ± 2.6 g of fat (72.2 ± 2.8 g/day [60], 58.2% predicted). Thus, in the combined adult and child PMD cohort, fat intake was significantly decreased at 30.2 ± 2.1 g/day (54.2% predicted).

In the adult (n = 13/22) and child (n = 15/38) PMD subjects with decreased fat calorie consumption (n = 28, defined as < 30% proportion of daily Kcal intake), GI symptoms were common. Dysphagia was reported in 13/28 (46.4%) subjects, constipation in 16/28 (57.1%), reflux in 10/28 (35.7%), and GI dysmotility in 8/28 (28.6%). A total of 11/28 (39.3%) subjects with decreased fat calorie consumption consumed > 60% of their daily calories as CHO, and 12/28 (42.9%) consumed > 20% of their total calories from PRO. Of the 13 subjects who reported dysphagia, decreased fat calorie consumption with corresponding high CHO calorie consumption was observed in 7/13 (53.8%), suggesting that GI symptoms are likely associated with altered macronutrient consumption in PMD.

Zweers et al. reported low PRO intake (1.1 ± 0.34 g/kg) in their adult PMD cohort compared to their corresponding population mean [25, 26]. In our study cohort, PRO intake was 2.0 ± 0.2 g/kg (217% predicted) and 0.60 ± 0.1 g/kg (118.5% predicted) in child and adult PMD subjects, respectively. Of note, % predicted PRO intake (g/day) decreases with age as PRO goals increase with age [32]. Indeed, males 14–18 years old in this cohort (n = 4) consumed only 55.9% predicted, while PRO intake in the younger age groups either met or exceeded the DRI PRO goals (Table e-10). The observation of high PRO intake in young children in the healthy general population is common, due to high consumption of dairy products [62, 63].

##### c. Altered Macronutrient Distribution Among Subjects Who Consumed ≤ 75% Predicted Calorie Intake

**Fig. 3:**
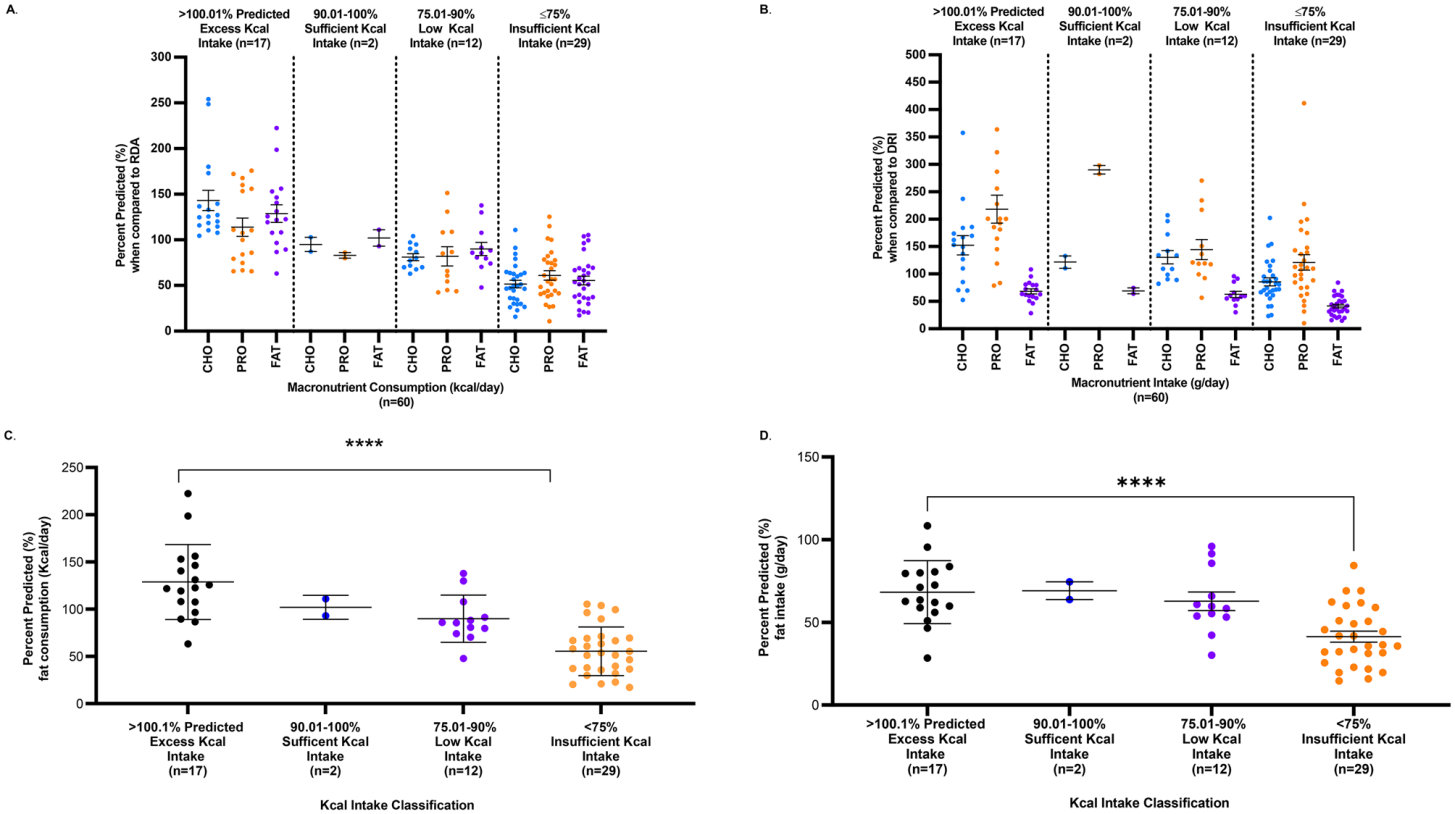
**A** Macronutrient consumption (Kcal/day) based on calorie intake classification. Subjects were classified by their daily Kcal intake, as either ‘Excess’ intake (≥ 100.1% predicted, n = 17), ‘Sufficient’ intake (90.01- 100% predicted, n = 2), ‘Low’ intake (75.01–90% predicted, n = 12), and ‘Insufficient’ intake (≤ 75% predicted, n = 29). Results show that the macronutrient consumption goal is met in the ‘Excess’ and ‘Sufficient’ Kcal groups, but is significantly reduced in the ‘Insufficient’ Kcal group, at 441.9 ± 38 kcal (51. 5% mean predicted values) for CHO, 184.5 ± 15.7 kcal for PRO (61.1% predicted), and 266.2 ± 20.2 kcal for fat (54.5% predicted) when compared to the other Kcal groups, *p* < 0.0001. **B** Macronutrient intake (g/day). Macronutrient consumption by subjects in the ‘Insufficient’ Kcal group (≤ 75.01% predicted intake) trends lower compared to the other Kcal groups, with significantly lower fat intake at 41.3% predicted, *p* < 0.0001 (ANOVA). **C** Percent (%) predicted fat calorie consumption. Fat calorie consumption (% predicted Kcal/day) was lowest in the ‘Insufficient’ Kcal intake group compared to the other groups, ****p < 0.0001(ANOVA, F(3,56) = 21.65). **D** Percent (%) predicted fat intake (g/day). Absolute fat intake (g/day) was significant decreased in the ‘Insufficient’ Kcal group (n = 30), ****p < 0.0001(ANOVA, F(3,56) = 9.298)

To assess whether the altered macronutrient intake was concordant with energy intake, we classified PMD subjects as having either ‘excess’ % predicted Kcal intake (≥ 100.01% Kcal, n = 17), ‘sufficient’ (90.01- 100% Kcal, n = 2), ‘low’ (75.01 – 90%, n = 12), or ‘insufficient’ intake (≤ 75% Kcal, n = 29) (Table e-11, Fig. 3A). Based on WHO-MOTIVATOR predicted energy intake, ‘insufficient’ energy intake (consumption of ≤ 75% predicted) was observed in 50% of adult PMD subjects (11/22). Of those, 4/11 (36.3%) consumed 50–70% of their predicted energy intake, while 7/11 (63.6%) consumed < 50% predicted. In child PMD subjects, ‘insufficient’ energy intake (≤ 75% predicted) was observed in 18/38 subjects (47.4%); 10/18 (55.6%) consumed 50–70% of their predicted energy intake; and 8/18 (44.4%) consumed < 50% predicted. These results emphasize the remarkably high prevalence of inadequate Kcal intake in our PMD cohort.

On comparing the mean % predicted value for each macronutrient consumption [Estimated macronutrient consumption (Kcal/day)/WHO-MOTIVATOR predicted Kcal intake (Kcal/day)/RDA predicted macronutrient distribution goal (%)], we observed a significant and gradual decline from the ‘Excess’ through to the ‘Insufficient’ Kcal groups for CHO (R^2^ = 0.56, *p* < 0.0001, n = 60), PRO (R^2^ = 0.32, *p* < 0.0001, n = 60), and fat (R^2^ = 0.58, p =  < 0.0001, n = 60). Macronutrient consumption goal was met in the ‘Excess’ and ‘Sufficient’ Kcal groups but was significantly inadequate in the ‘Insufficient’ Kcal group, at 441.9 ± 38.0 kcal for CHO (51.5% mean predicted values), 184.5 ± 15.7 kcal for PRO (61.1% predicted), and 266.2 ± 20.2 kcal for fat (54.5% predicted) when compared to the other Kcal groups, *p* < 0.0001 (ANOVA, Table e-11, Fig. 3A and C). Thus, the decreased macronutrient consumption likely accounts for the overall low energy intake in the ‘Insufficient’ Kcal group.

A similar observation was made when macronutrients were expressed as g/day and compared across the four Kcal intake groups, for CHO (R^2^ = 0.24, *p* < 0.0001, n = 60), PRO (R^2^ = 0.24, p = 0.0001, n = 60), and fat (R^2^ = 0.41, *p* < 0.0001, n = 60). Most notably, fat intake was lowest in the ‘Insufficient’ Kcal group when compared to the other groups, at 41.3 ± 3.3% predicted (29.6 ± 2.2 g/day, Table e-12, Fig. 3B and D, *p* < 0.0001 (ANOVA).

#### Daily Calorie Consumption in BMI Groups is Not Predictable

Healthy adults with either very low (< 801 kcal/day) or low energy intake (≤ 1,000 kcal/day), tend to suffer medical complications, including a wide host of concerns, such as poor bone health, decubitus ulcers, intensifying gastroparesis, and vitamin and mineral deficiencies [55].

**Fig. 4:**
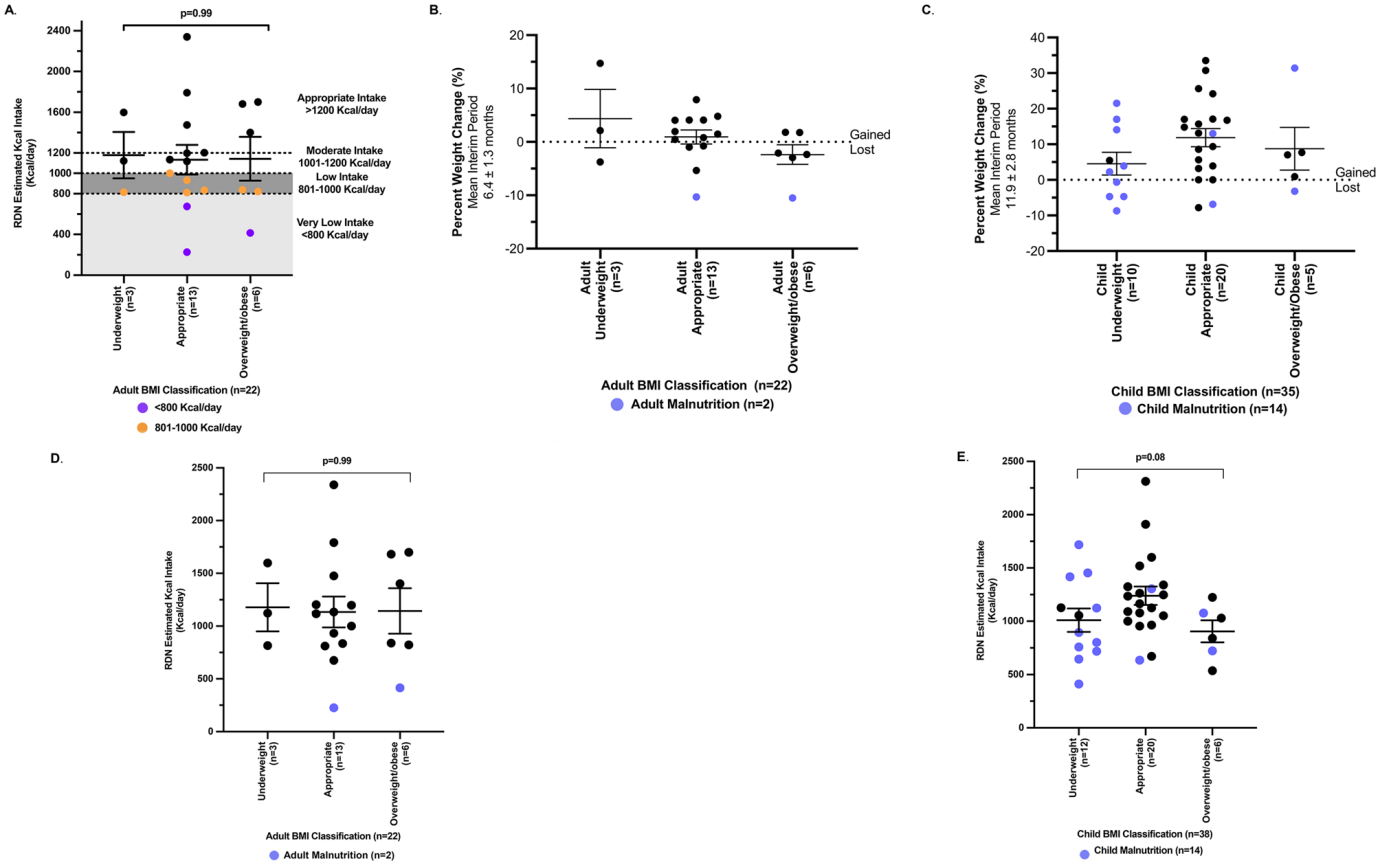
**A** Comparison of BMI and daily Kcal intake in adult subjects only (n = 22). Mean daily Kcal intake in the underweight (1,179 ± 227.7 kcal/day, n = 3), appropriate (1,134 ± 145.4 kcal/day, n = 13), and overweight/obese (1,143 ± 215.5 kcal/day, n = 6) adult subjects (n = 22) was not significantly different, p = 0.99, ANOVA. Subjects with low Kcal intake (801–1000 kcal/day) are indicated in orange, while subjects with very low Kcal intake (< 800 kcal/day) are indicated in purple. **B** Percent weight change (%) over mean interim period of 6.4 ± 1.3 months in adult subjects (n = 22). There were a total of 9/22 (40.9%) adult subjects who lost weight and 13/22 (59.1%) who gained weight. There were 2 subjects who met ASPEN and/or GLIM malnutrition criteria, one in the appropriate BMI group and one in the overweight BMI group, subjects are indicated in purple. **C** Percent weight change (%) over mean interim period of 11.9 ± 2.8 months in child subjects (n = 35)*. There were a total of 7/35 (20%) child subjects who lost weight and 28/35 subjects (80%) who gained weight. There were 14 subjects who met ASPEN malnutrition criteria, subjects are indicated in purple. *There were n = 3/38 subjects with no weight change observed in the interim period between prior clinic visits and RDN baseline assessment. **D** Malnutrition classification in adult PMD subjects (n = 22) and daily Kcal intake. There were 2/22 (9.1%) adult subjects who met malnutrition criteria, both subjects meeting ASPEN and GLIM criteria, subjects with malnutrition are indicated in purple. Mean daily Kcal intake in the underweight (1,179 ± 227.7 kcal/day, n = 3), appropriate (1,134 ± 145.4 kcal/day, n = 13), and overweight/obese (1,143 ± 215.5 kcal/day, n = 6) adult subjects (n = 22) were not significantly different, p = 0.99, ANOVA. **E** Malnutrition classification in child PMD subjects (n = 38) and daily Kcal intake. There were 14/38 (36.8%) child subjects who met ASPEN malnutrition criteria. Malnutrition is present in each BMI category, indicated in purple. Mean RDN-estimated Kcal intake (Kcal/day) in underweight child subjects (n = 12) was 1,010 ± 109.8 (SEM) Kcal/day, 1,240 + 86.4 kcal/day in subjects with appropriate BMI (n = 20), and 905.1 ± 103.3 kcal/day in overweight/obese (n = 6) subjects. Estimated Kcal intake was not significantly different between the three BMI groups, p = 0.08, ANOVA

Mean daily Kcal intake in the underweight (n = 3), appropriate (n = 13), and overweight (n = 6) adult subjects (n = 22) was not significantly different, p = 0.99, ANOVA (Fig. 4A). Patient-reported GI symptoms were prevalent in all 3 BMI groups. Of the PMD adult subjects with appropriate BMI (n = 13/22, 59.1%), 2 subjects (15.4%) consumed < 800 (‘very low’) Kcal/day, 3 (23.1%) consumed 801–1,000 (‘low') Kcal/day, 4 (30.8%) consumed 1,001- 1,200 (‘moderate’) Kcal/day, and 4 (30.8%) consumed > 1,200 (‘appropriate’) Kcal (Table e-13, Fig. 4A). Underweight PMD adults (n = 3/22, 13.6%) consumed ‘low’ (n = 1) or ‘appropriate’ (n = 2) Kcal/day consumption. Of the 6 adults classified as overweight/obese, 1 consumed ‘very low’ Kcal/day, 2 consumed ‘low’ Kcal/day, and the remaining 3 adults consumed > 1,200 kcal/day, at a mean of 1,595 ± 96.5 kcal/day, which is within a calorie range (1,400–1,700 kcal/day) known to contribute to weight loss in obese adults [55] (Table e-13, Fig. 4A). These data demonstrate the lack of association between calorie consumption and BMI in our PMD cohort, and that malnutrition (≤ 75% predicted Kcal intake) can be present in the context of appropriate BMI.

Across the total adult and children PMD cohort, 11/60 (18.3%) subjects were overweight/obese. Of those, 6/11 (54.5%) gained an average of 5.7 ± 3.4 kg (range, 0.2- 22.2 kg) across a mean interim period of 11.6 months, despite their low mean Kcal intake of 909.8 ± 84.3 kcal/day (Fig. 4B–C). In summary, these results highlight that BMI should not be considered as the sole indicator of nutritional status in PMD. However, as this was a cross-sectional study, monitoring longitudinal BMI and growth in a future PMD study to characterize the nutritional status of PMD subjects would be critical.

#### Weight Loss was Observed in 16/60 (26.7%) PMD Subjects at Baseline RDN Visit

Having recognized that insufficient energy consumption occurred across our PMD cohort, we next explored weight trajectory, by comparing weight measured at the RDN baseline evaluation to a prior weight measurement documented in the electronic medical record at a physician visit up to ~ 12 months prior. We found a total of 16 subjects, consisting of 9 adults and 7 pediatric subjects, who presented with weight loss. At a mean interim period of 8.8 ± 1.5 months, mean weight loss was observed to be -2.4 ± 0.5 kg (mean ± SD, -4.8 ± 0.8%, range, -7.7 to -0.2 kg). When analyzed by age, mean weight loss for adults (n = 9) was -2.8 ± 0.8 kg (-4.3 ± 1.2%, range, -7.7 to -0.5 kg, mean interval period, 6.4 ± 1.3 months) and for children (n = 7) was -1.8 ± 0.5 kg (-5.4 ± 1.1%, -3.4 to -0.2 kg, 11.9 ± 2.8 months) (Table e-14, Fig. 4B–C). These child subjects had a preceding weight for age z-score of -2.7 ± 4.1 (range, -10.5 to 1.8, median, -1.2), height for age z-score of -1.2 ± 2.2 (range, -4.7 to 1.4, median, -1.1), and mean BMI z-score of -0.7 ± 1.8 (range, -3.2 to 1.5, median, -0.7). At the time of baseline nutritional assessment, mean weight for age z-score was -4.0 ± 5.0 (-13.6 to 1.4, median, -1.2), height for age z-score -2.2 ± 2.9 (-4.6 to 1.2, median, -1.1), and mean BMI-Z score -2.6 ± 2.8 (-6.2 to 1.1, median, -0.8). Thus, the mean difference (∆) in z-scores between the 2 clinic visits was -1.3 ± 0.9 z-scores for weight for age (Table e-14), -1.0 ± 1.1 z-scores for height for age, and -1.8 ± 1.0 z-scores for BMI across a mean interim period of 8.8 ± 1.5 months.

Of the 16 adult and child subjects with weight loss, 9/16 (56.3%) reported dysphagia, 5/16 (31.3%) reported GI dysmotility, and 4/16 (25%) reported loss of appetite (Table e-15). In terms of Kcal consumption, 9/16 (56.3%) consumed ≤ 75% predicted Kcal/day (mean of 670.1 ± 99 kcal/day), and 4/16 (25%) consumed 75–100% predicted Kcal/day (1,400 ± 164.7 kcal/day). Most obese individuals will lose weight when they consume between 1,000 – 1,500 kcal/day [55]. In these subjects with weight loss, 5/16 were in the overweight/obese BMI category, while 5/16 were underweight and 6/16 had appropriate BMI (Table e-14).

#### Malnutrition was Prevalent in 16/60 (26.7%) PMD Subjects

As 29/60 (48.3%) PMD subjects in this study consumed ≤ 75% predicted WHO-MOTIVATOR calorie intake, we proceeded to evaluate for malnutrition. Malnutrition is defined by the WHO as deficiencies, excesses, or imbalances in a person’s intake of energy and/or nutrients and includes those in the groups of undernutrition, micronutrient-related malnutrition, and overweight/obesity [38]. Unexpectedly, we identified a total of 16/60 (26.7%) subjects who met criteria for malnutrition. These were not the exact same 16/60 subjects with weight loss described earlier. Of the adult subjects, 2/22 (9.1%) had malnutrition, both of whom met the GLIM [39] criteria for malnutrition based on weight loss and/or low BMI, reduced food intake, and AND/ASPEN [37] criteria of reduced calorie intake of ≤ 75% predicted for > 30 days and percent (%) weight loss (Table e-16–e-17, Fig. 4D). In the child cohort, 14/38 subjects (36.8%) met the AND/ASPEN criteria for malnutrition based on BMI z-score and/or inadequate nutrient intake alone [40]. 1/14 child cohort subjects had mild malnutrition (BMI z-score -1.0 to -1.99 with 51–75% predicted calorie intake), 7/14 had moderate (BMI z-score—2.0 to -2.99 or 26–50% predicted calorie intake), and 6/14 had severe malnutrition (BMI for age z-score ≤ -3 or more or ≤ 25% predicted calorie intake) [40] (Tables e-17, e-18, Fig. 4E). Among 6/24 (25%) subjects with malnutrition who had a G-tube, 5 were children and 1 was an adult. Genetic etiologies of subjects with malnutrition are outlined (Table e-16).

#### Significantly Decreased Fluid Intake

Mean daily fluid intake was 1,704 ± 199.6 ml (76.1% predicted fluid intake using the Holliday Segar method) [33] and 1,128 ± 91.0 ml (77.3% predicted) in adult (n = 16) and child (n = 29) subjects, respectively (Table e-19). In addition, fluid intake decreased from the ‘excess’ to ‘insufficient’ Kcal intake groups, at 70.8 ± 9.0% predicted p = 0.62, ANOVA (Table e-12).

Of the 8 subjects with a G-tube, fluid intake per dietary history was able to be assessed in 7 subjects (6 pediatric; 1 adult). All 7 subjects met their goal fluid intake at a mean 105.8% of predicted. Fluid intake was significantly higher in subjects with a G-tube (1,659 ± 232.0 ml/day; 105.8% predicted, n = 7) compared to those without a G-tube who consumed (1,265 ± 105.6 ml/day; 70.5% predicted, n = 36) (p = 0.01). Due to the increased osmolality and the increased renal solute load imposed by PMD mitochondrial supplements, extra fluid above the goal reference may be indicated. Therefore, the observation of insufficient fluid intake in this PMD cohort is a concern that needs to be addressed.

#### Correlation of Nutritional Intake to Objective Muscle Measurements and Survey Assessments

In order to assess the clinical meaning of the nutritional parameters, we assessed the correlation of daily Kcal intake, daily CHO, PRO, and fat calories (g/day and % daily Kcal) as percent (%) predicted values, and weight loss (%) (Tables e-22 and e-23) to myopathy objective measures and survey responses that are routinely administered in our clinic (Table e-24). Briefly, objective MM-COAST [28] measurements of muscle strength using hand-held dynamometry, 30 second Sit-to-Stand test (30s STS) and 6-minute walk test (6MWT) for exercise intolerance, and dynamometry repetitions for muscle fatigue were able to be completed in clinic alongside baseline nutrition evaluations in 33/60 (55%) subjects (Table e-20, Supplemental Fig. 2). MM-COAST assessments in 31 subjects were previously reported in Flickinger et al. [28]. Assessments in this study cohort (n = 33) redemonstrated the prevalence of muscle weakness, exercise intolerance, and muscle fatigue typical of PMD (Supplemental Fig. 2). Mean MM-COAST Composite Score of all objective assessments was 1.43 ± 0.13 (SEM, n = 33), which indicates a high PMD disease severity [28]. Subjects were invited to complete patient-reported survey assessments of fatigue, quality of life (QoL), and performance (Table e-22), as part of a larger study (MacMullen, et al., unpublished). In this cohort, the Pediatric Quality of Life Inventory (PedsQL, n = 37) [50], which has also been validated in adults [64], Modified Fatigue Impact Scale (MFIS, n = 33) [51], Karnofsky/Lansky Performance Scale (K/L, n = 37) [52], and Pediatric Evaluation of Disability Inventory Computer Adaptive Test (PEDI-CAT, n = 11) [53] revealed impacted physical health quality of life, fatigue, effortful activity, and low mobility function, respectively (Supplemental Fig. 3).

**Fig. 5:**
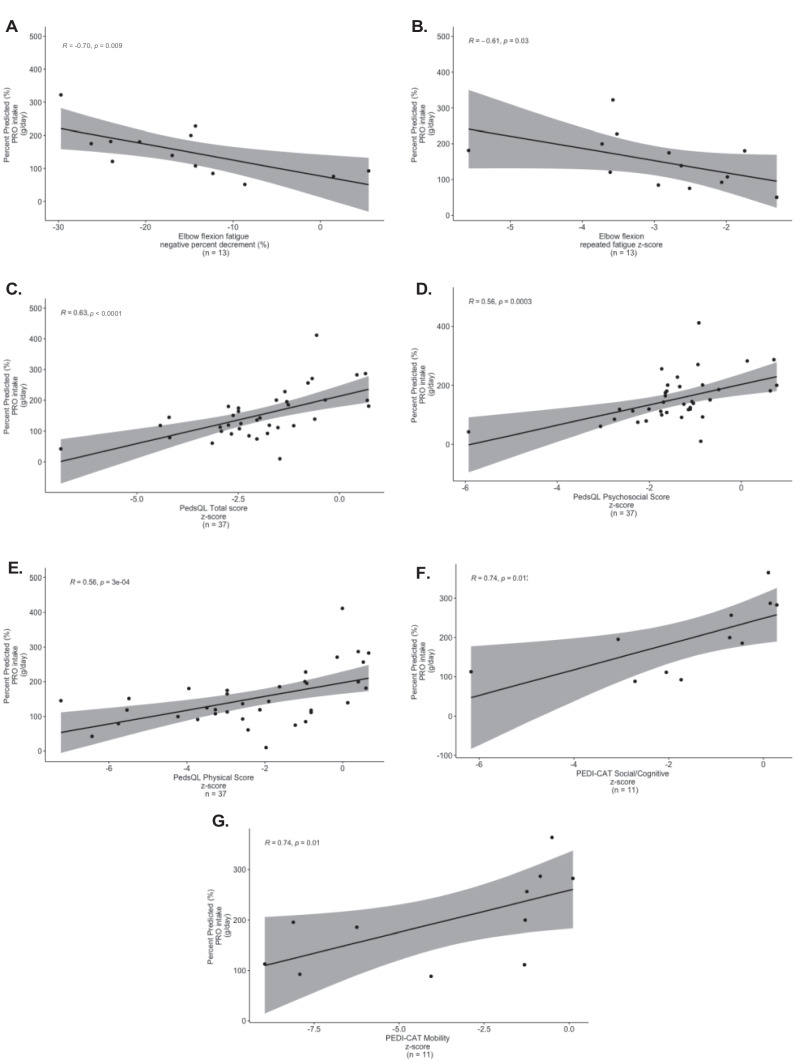
**Correlations of objective assessments and surveys to protein (PRO) intake (g/day) for all subjects (n = 60). A** Elbow flexion fatigue negative percent decrement (%) to percent predicted (%) PRO intake (g/day). There was a strong negative correlation between elbow flexion fatigue negative percent decrement to PRO intake (g/day) indicating decreased muscle fatigue with increased PRO intake (r = -0.70, p = 0.009, n = 13). **B** Elbow flexion 6th repetition fatigue z-score/percent predicted (%) PRO intake (g/day). There was a strong negative correlation between elbow flexion 6th repetition fatigue z-score and PRO intake (g/day) (r = -0.61, p = 0.03, n = 13), which demonstrates decreased muscle fatigue with increased PRO intake. **C** PedsQL total score z-score to percent predicted (%) PRO intake (g/day). There was a moderate positive correlation between PedsQL total z-score and PRO intake (g/day) (r = 0.63, *p* < 0.0001, n = 37). **D** PedsQL psychosocial score z-score to percent predicted (%) PRO intake (g/day). There was a moderate positive correlation between PedsQL psychosocial score z-score and PRO intake (g/day) (r = 0.56, p = 0.0003, n = 37). **E** PedsQL physical score z-score to percent predicted (%) PRO intake (g/day). There was a moderate positive correlation between PedsQL physical score z-score and PRO intake (g/day) (r = 0.56, p = 0.0003, n = 37). **F** PEDI-CAT social/cognitive z-score to percent predicted (%) PRO intake (g/day). There was a strong positive correlation between PEDI-CAT social/cognitive z-score and PRO intake (g/day) ( r = 0.74, p = 0.01, n = 11). **G** PEDI-CAT mobility z-score to percent predicted (%) PRO intake (g/day). There was a strong positive correlation between PEDI-CAT mobility z-score and PRO intake (g/day) (r = 0.74, p = 0.01, n = 11)

**Fig. 6:**
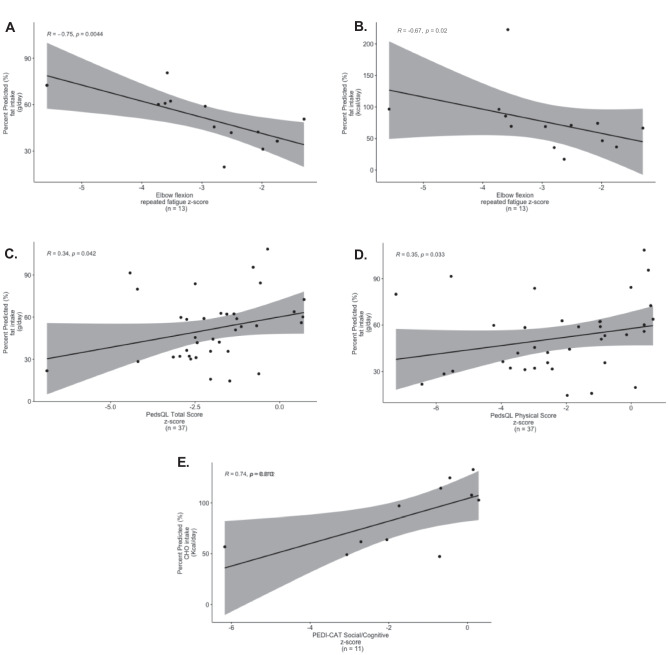
**A, C, D** Correlations of objective assessments and surveys to fat and CHO intake (g/day) for all subjects (n = 60) Fig. 6B, E Correlations of objective assessments and surveys to macronutrient consumption (% Kcal) for all subjects (n = 60). **A** Elbow flexion 6th repetition fatigue z-score/percent predicted (%) fat intake (g/day). There was a strong negative correlation between elbow flexion 6th repetition fatigue z-score and fat intake (g/day) (r = -0.75, p = 0.004, n = 13), which demonstrates decreased muscle fatigue with increased fat intake. **B** Elbow flexion 6th repetition fatigue z-score/percent predicted (%) fat consumption (Kcal/day). There was a moderate negative correlation between elbow flexion 6th repetition fatigue z-score and fat consumption (Kcal/day) (r = -0.67, p = 0.02, n = 13), which demonstrates decreased muscle fatigue with increased fat calorie consumption. **C** PedsQL total score z-score/percent predicted (%) fat intake (g/day). There was a weak positive correlation between PedsQL total score z-score and fat intake (g/day) (r = 0.34, p = 0.04, n = 37). **D** PedsQL physical score z-score/percent predicted (%) fat intake (g/day). There was a weak positive correlation between PedsQL physical score z-score and fat intake (g/day) (r = 0.35, p = 0.03, n = 37). **E** PEDI-CAT social/cognitive z-score to percent predicted (%) CHO consumption (Kcal/day). There was a strong positive correlation between PEDI-CAT social/cognition z-score and CHO calorie consumption (r = 0.74, p = 0.012, n = 11)

**Fig. 7:**
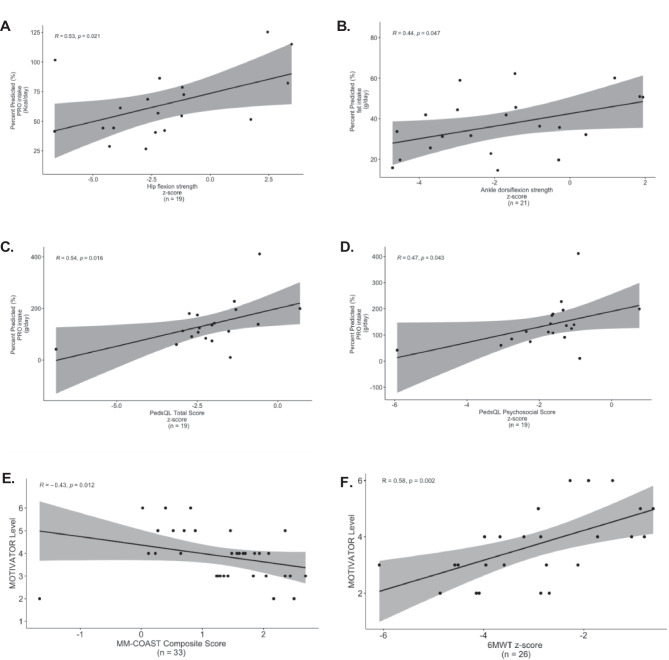
**Correlations of objective assessments and surveys to macronutrient consumption (g/day and Kcal/day) in subjects with inadequate Kcal intake (≤ 75% predicted) (n = 29)**. **A** Hip flexion strength z-score/percent predicted PRO consumption (Kcal/day). There was a moderate positive correlation between hip flexion strength z-score and PRO calorie consumption (Kcal/day) (r = 0.53, p = 0.021, n = 19). **B** Ankle dorsiflexion strength z-score/percent predicted (%) fat intake (g/day). There was a moderate positive correlation between ankle dorsiflexion strength z-score and fat intake (g/day) (r = 0.44, p = 0.047, n = 21). **C** PedsQL total score z-score/percent predicted (%) PRO intake (g/day). There was a moderate positive correlation between PedsQL total score z-score and PRO intake (g/day) in 19/29 (r = 0.54, p = 0.016, n = 19). **D** PedsQL physical score z-score/percent predicted (%) PRO intake (g/day). There was a moderate positive correlation between PedsQL psychosocial score z-score and PRO intake (g/day) (r = 0.47, p = 0.043, n = 19). **E** MOTIVATOR Level/MM-COAST Composite Score. There was a moderate negative correlation between MOTIVATOR Level and MM-COAST Composite Score (R = -0.43, p = 0.012, n = 33). **F** MOTIVATOR Level/6-minute walk test z-score. There was a moderate positive correlation between MOTIVATOR Level and 6 Minute Walk Test z-score (R = 0.58, p = 0.002, n = 26)

In terms of correlations to objective measurement of muscle strength across the full cohort, there was a moderate negative correlation between PRO intake (g/day, r = -0.70, p = 0.009, n = 13, Fig. 5A) and PRO calorie consumption (Kcal/day, r = -0.82, p = 0.001, n = 13, Table e-23) with elbow flexion % decrement indicating decreased muscle fatigue with increased PRO intake. There was also a moderate negative correlation between PRO (r = -0.61, p = 0.03, p = 13) and fat (r = -0.75, p = 0.004, n = 13) intake (g/day) and fat calorie consumption (r = -0.67, p = 0.02, n = 13) with elbow flexion 6th repetition z-scores, also indicating decreased muscle fatigue (Figs. 5B and 6A–B). Further, there was a strong positive correlation between PRO intake (g/day, r = 0.63, *p* < 0.0001, n = 37) and PedsQL Total function z-score, with specific correlations to the subsections of psychosocial function (r = 0.56, p = 0.003, n = 37) and physical function (r = 0.56, p = 0.0003, n = 37); and a weak positive correlation between PRO calorie consumption (Kcal/day) and PedsQL school/work function (r = 0.39, p = 0.049, n = 26) (Tables e-22–e-23). There was also a strong positive correlation between PRO intake (g/day) and PEDI-CAT mobility z-scores (r = 0.74, p = 0.01, n = 11) and social/cognitive z-scores (r = 0.74, p = 0.01, n = 11). PRO calorie consumption (Kcal/day) correlated with the PedsQL school/work function z-score (r = 0.39, p = 0.049, n = 26). When analyzed in the ‘Insufficient’ (≤ 75% Kcal intake) group, (Tables e-25–e-26, Fig. 7A), there was a moderate positive correlation between PRO calorie consumption (Kcal/day) and dominant hip flexion strength (r = 0.53, p = 0.02, n = 19).

Across the cohort, there was a strong negative correlation between elbow flexion 6th repetition fatigue z-scores and fat intake (g/day) (r = -0.75, p = 0.004, n = 13) and fat calorie consumption (Kcal/day) (r = -0.67, p = 0.02, n = 13), indicating decreased muscle fatigue with fat consumption (Fig. 6A–B). Fat intake (g/day) was weakly correlated with improved PedsQL total score (r = 0.34, p = 0.04, n = 37) and its physical function subsection (r = 0.35, p = 0.03, n = 37). In the ≤ 75% Kcal intake group, fat intake (g/day) positively correlated with dominant ankle dorsiflexion strength (r = 0.44, p = 0.047, n = 21, Fig. 7B). PRO intake (g/day) moderately correlated with both PedsQL total function z-score (r = 0.54, p = 0.02, n = 19) and PedsQL psychosocial function z-score (r = 0.47, p = 0.04, n = 19, Table e-25, Fig. 7C–D). These results collectively suggest improved muscle fatigue and quality of life with increased PRO and fat calorie consumption and absolute intake. Specifically, in those with insufficient daily Kcal intake, improved muscle strength at hip flexion and ankle dorsiflexion was observed (Fig. 7A–B). Meanwhile, CHO calorie consumption positively correlated with PEDI-CAT social/cognition z-score (r = 0.74, p = 0.01, n = 11) across the cohort. There was no significant correlation between nuclear or mtDNA genetic etiologies and nutritional parameters.

In addition, there was a strongly positive correlation between PEDI-CAT social/cognition z-score (r = 0.78, p = 0.006, n = 11, Table e-24) with % predicted daily Kcal intake; and a strong negative correlation between percent (%) weight loss and hip flexion strength (r = -0.71, p = 0.018, n = 11), indicating the association of weight loss with decreased strength. On the other hand, a significant correlation was not observed between daily Kcal intake and the MM-COAST Composite Score (n = 33), p = 0.7. The MM-COAST Composite Score represents disease severity, and is a sum and average of test scores across five MM domains [28] which likely explains the observed lack of correlation in a relatively small cohort of subjects with MM-COAST Composite Scores (n = 33) in this study.

In summary, our results indicate that PRO and fat calories and/or absolute intake (g/day) were positively associated with improved muscle fatigue, quality of life, and specifically in those with insufficient daily Kcal intake (≤ 75% predicted), improved large-muscle group strength (hip flexion and ankle dorsiflexion). Similarly, higher CHO intake and daily Kcal intake were positively associated with improved QoL. We postulate that the correlations of macronutrients to improved myopathy measures and QoL are not specific to the macronutrient, but reflect the overarching need for increased daily Kcal intake in PMD.

Lastly, we evaluated the association of the MOTIVATOR activity levels (levels 1 through 6, Tables e-7–8) to the MM-COAST Composite Score in order to show that distinct AF levels as defined in the MOTIVATOR, depending on the mobility and fatigue status of an individual with PMD, aligns with the objective MM-COAST assessments of myopathy. A higher MM-COAST Composite Score indicates increased disease severity [28]. Indeed, there was a moderate negative correlation between MOTIVATOR levels and MM-COAST Composite Score (r = -0.43, p = 0.012, n = 33, Fig. 7E). This illustrates that lower MOTIVATOR levels with decreased mobility and increased muscle fatigue are associated to a higher PMD disease severity, supporting the clinical relevance and criterion validity of the MOTIVATOR. MOTIVATOR levels also positively correlated to the 6MWT z-score assessment of exercise intolerance (r = 0.58, p = 0.002, n = 26, Fig. 7F) to demonstrate that MOTIVATOR classification of fatigue (Tables e-7–8) is indeed representative of objective 6MWT fatigue assessments.

## Discussion

This is the first study to describe daily Kcal (energy) and macronutrient intake in a genetically-confirmed PMD cohort that includes both adults and children. Results of our study highlight the inadequacy of PMD subjects across the age span in meeting fundamental nutritional needs. Specifically, results from this study revealed significantly low daily Kcal intake (Table e-5), low fat intake (g/day) (Table e-10), significantly decreased fluid intake (Table e-19), high prevalence of GI symptoms (Tables e-3, 15, 17, 18), lack of association of calorie intake to BMI (Table e-13), and the prevalence of malnutrition in 16/60 subjects (26.7%, Table e-16). In addition, higher PRO and fat intake correlated with improved muscle strength in those with insufficient daily Kcal intake (≤ 75% predicted); higher PRO and fat intake correlated with decreased muscle fatigue; and PRO, fat, and CHO intake correlated with improved QoL across the full PMD cohort (Tables e-22–26). Most notably, determination of the nutritional needs in PMD requires an understanding of PMD phenotype. To this end, we proposed a standardized approach to predict energy needs in PMD, by using a novel, customized scoring system named the MOTIVATOR.

The clinical phenotype of PMD is broad and multi-systemic. The most common symptoms of PMD include muscle weakness, fatigue, exercise intolerance, and imbalance [2], which may alter nutritional needs due to impaired motor function and mobility. In addition, reduced muscle mass [55] needs to be accounted for, as this decreases nutritional needs. Some PMD patients have strong muscle strength yet display low endurance; others present with significant ataxia affecting the quality of gait and/or extrapyramidal movements that are associated with gross motor inefficiency and thus high energy expenditure [65, 66]. Gastrointestinal symptoms are also very common in PMD [2], which may directly affect energy consumption and intestinal absorption. Commonly prescribed medications in PMD may have direct impact on nutritional needs. Thus, a comprehensive understanding of a PMD individual’s phenotype, including mobility and activity levels (sedentary or active) and a review of medications, is essential in order to estimate energy needs (Supplemental Fig. 1).

Indirect calorimetry (IC) is considered the gold standard approach to measure REE. REE represents the energy expended during a 24 hour non-active period to maintain involuntary functions, such as respiration, cardiac output, and body temperature regulation [67]. In healthy sedentary adult subjects, REE constitutes two-thirds of the total energy expenditure [31] and is closely related to lean body mass, which is more metabolically active than fat mass. Notably, differences in lean body mass account for approximately 80% of the variance in measured REE [55]. IC would also provide a respiratory quotient [68], indicating which macronutrient(s) are chiefly oxidized. However, IC is not broadly accessible.

REE prediction equations, such as the Food and Agriculture Organization/WHO equations [32], are used in conjunction with an AF multiplied by the REE to calculate total energy estimation [32, 55, 56]. The WHO equations include body weight, are stratified per age group, and take changes in body composition across the lifespan into account. The HBE [36] and the Mifflin St Jeor (MSJ) [57] are REE predictive equations that are commonly used in adults and incorporate height and weight into the calculation. As previously published studies of adult cohorts frequently utilized the HBE and MSJ equations, we compared the WHO, HBE, and MSJ equations and found the calculated REE to be statistically comparable (Table e-4), thus supporting our proposal to utilize the WHO predictive equation across all ages in our PMD cohort. We recognize that a major limitation to utilizing predictive equations is the assumption that these equations are appropriate for PMD.

In an attempt to ascertain the reliability of predictive equations, prior studies have been conducted to compare indirect calorimetry (IC) to REE prediction equations in various PMD patient cohorts. A study of child PMD subjects (n = 20 PMD; n = 27 matched controls) reported REE to be 1,050.5 ± 229.0 (mean ± SD) Kcal/day in PMD and 1,022 ± 52.6 kcal/day in control subjects, p = 0.086 [68]. When comparing the predictive capacity of the Schofield equation for REE (1,053.8 ± 221.8 kcal/day) to the Food and Agriculture Organization/World Health Organization/United Nations University (FAO/WHO/UNU) equation (1,056.4 ± 210.5 kcal/day), Fiuza-Luces et al. found no significant difference between the predictive equations (p = 0.67) nor a significant difference between the predictive equations and IC-measured REE (p = 1.00) [68]. Nevertheless, the authors noted that predictive equations may inconsistently estimate REE, indicating that IC remains the ideal approach to measurement of REE in PMD. Of note, the WHO-REE in the child PMD subjects in our study cohort (n = 38) was estimated to be 1,037 ± 340.9 kcal/day, which was comparable to the IC-measured REE in the Fiuza-Luces et al. study of 1,050.5 ± 229.0 kcal/day (n = 20) [68].

In a study performed by Zweers et al. [69], REE was measured in PMD adults (harboring m.3243A > G) and healthy subjects by IC and compared to 21 predictive equations. Results showed that IC-REE in PMD subjects was 1,430 ± 221 kcal (mean ± SD, n = 38), which trended lower than in healthy counterparts at 1,532 ± 182 kcal (n = 25), p = 0.052. Additionally, of the 21 REE predictive equations, the 3 most reliable equations for predicting REE in PMD subjects included the Henry (based on height and weight, 76% of PMD patients with accurate REE estimation), the Harris-Benedict (71%), and the Muller (based on fat-free mass, 71%) [69]. In comparison, mean WHO-estimated REE in our PMD adult cohort was 1,389 ± 206.5, n = 22. As results are comparable, this suggests that implementation of WHO-predicted REE estimates would be appropriate in PMD, although we would need to confirm this by direct measurement of IC-REE in this cohort. Indeed, future studies to assess IC-measured REE would be crucial in defining REE needs specific to each PMD individual in clinical trials involving nutritional modifications, and to determine the impact of systemic adaptation to energy repletion with nutritional support.

The ratio of TEE to REE is used to define the physical activity level (PAL). In this one study, the authors measured PAL [69], which we refer to as AF in this study, to be 1.4 ± 0.24 (mean ± SD) using a multisensor actometer (SenseWear), and thus concluded that PAL should be measured by accelerometry or to apply a standard AF of 1.4 for estimation of TEE in PMD. While we would agree to an AF of 1.4 for PMD adults with moderate activity without fatigue (Table e-1), we would not recommend a common AF across all PMD subjects, as broad variability in fatigue and mobility levels resulting in impaired functional performance among PMD subjects is observed [28]. Moreover, Apabhai et al. [70] found that half of their adult PMD cohort had an average daily expenditure of less than 1.4 times their REE due to low levels of physical activity. It is also worth noting that chronic Kcal insufficiency may be associated with an adaptive decline in physical activity levels [71].

Indeed, the most frequent MOTIVATOR-AF applied to 8/22 (36.4%, range 0.8 -1.4) in our adult PMD cohort was 1.1, followed by 1.0 and 1.2, each in 5/22 (22.7%) subjects, and 1.3 in 3/22 (13.6%). In our child PMD subjects (n = 38), where play and physical activity is higher as compared to adults [72], we still observed a broad range in the AFs applied (0.8—1.7), where the most frequently applied MOTIVATOR-AF was 1.4 in 9/38 (23.7%) subjects, followed by 1.1, 1.3, and 1.7, each in 8/38 (21.1%) subjects. Thus, while our WHO-MOTIVATOR estimated TEE was 1,554 ± 58.0 kcal (Table e-5) in adult PMD subjects (n = 22), their PMD cohort had a higher TEE of 1,985 ± 243 kcal/day using a fixed AF of 1.4, and a TEE of 2,058 ± 414 kcal/day (n = 38) when an actometer was used [69]. Concerns regarding accuracy of actometer measurements depending on the physical activity [73, 74] and individual reporting of PAL [69] have been raised. Further, overestimation of energy expenditure with the SenseWear has been reported [75, 76]. By contrast, the MOTIVATOR facilitates a standardized approach to customized estimation of a more reliable energy goal that better approximates the PMD cohort gross motor phenotype. One limitation is that the MOTIVATOR has not been compared to gold standard methods of estimating TEE using IC [77], which we will seek to address in future studies.

We included two infants in the pediatric cohort. WHO equations are validated for age ranges 0–3 years, but estimation of AF in infants is challenged by the fact that growth is a larger component of energy requirement in early life [32]. As energy expenditure in infants is prioritized for growth more than activity, the RDA [32] was utilized to estimate energy needs in the 2 infants in this study. Subsequently, we established a lower age limit of 1 year for use of the MOTIVATOR.

We did not identify an association between daily Kcal intake and BMI, where underweight (n = 3), appropriate weight (n = 13), and overweight/obese adult subjects (n = 6) consumed mean Kcal of 1,179 ± 227.7, 1,134 ± 145.4, and 1,143 ± 215.5, respectively (Table e-13), suggesting dysregulation of body weight in PMD regardless of energy intake. This highlights how individualized PMD energy needs are, as most individuals would lose weight if their energy intake was indeed inadequate. To this point, most healthy adults maintain relatively stable body weight over time, and adjust energy intake in response to changes in energy requirements to maintain an energy balance [78]. In our study cohort, 16/60 subjects, 26.7% (9 adults, 7 child subjects) lost weight (-2.4 ± 0.5 kg, mean, SEM) and with mean Kcal intake of 978.3 ± 112.5 kcal/day (69.5% predicted) over an interim period of up to 11.9 months between initial nutrition assessment and preceding weight obtained from the electronic medical records. Of note, 8/16 subjects with weight loss overlapped with the malnutrition cohort (n = 16), thus 8/16 (50%) of subjects with malnutrition did not have weight loss. Conversely, among the overweight/obese individuals across the PMD cohort (n = 12), 5 subjects lost weight, while 7 subjects gained weight (5.5 ± 7.5 kg), despite having a low mean Kcal intake of 909.8 ± 222.9 kcal/day. These results emphasize the importance of conducting a comprehensive nutritional assessment (Supplementary Fig. 1) to avoid the pitfalls of ascertaining nutritional status based on BMI alone. These results also serve as a reminder that not all calories are equal [79]. However, it would be important to highlight that a limitation of comparing height and weight measurements from the EMR is that different scales, clothing, and methods used across different clinics may contribute to the variability. Further, inaccuracies in obtaining height and weight measurements is a well-recognized challenge [80–82].

An unexpected finding in this study was the prevalence (26.7%) of malnutrition among our PMD subjects (Table e-16). Prolonged under- or malnutrition can contribute to recurrent infections and dehydration, leading to headaches and dizziness in the general population [83]. These symptoms are also prevalent in PMD, and therefore malnutrition symptoms can be overlooked in PMD. In this study, diagnosis of malnutrition in adult PMD subjects (n = 2/22) was based on weight loss or inadequate intake and BMI; and child PMD (n = 14/38) malnutrition was diagnosed based on inadequate intake and/or BMI (Tables e-17 and e-18). In future studies, additional indicators, such as weight gain velocity (≤ 2 years of age), deceleration in weight for length/height for age in children, and hand grip strength in adults should be considered, as an even higher prevalence of malnutrition in PMD would likely be captured.

We observed that PMD subjects can present with a BMI reflecting normal weight, overweight, or obesity, and be undernourished (malnutrition, Table e-16). Indeed, in the general population, overweight/obese individuals can be undernourished as a result of overconsumption of high-calorie, low-nutrient foods [84]. Of the adult and child PMD subjects, 48.3% (29/60) presented with chronic inadequate energy intake (≤ 75% daily Kcal) and were not previously treated with nutrition support. It was common for adult PMD subjects to report that in the absence of weight loss, their physician(s) did not address their nutritional status. We propose regular nutrition assessments at intervals of 3–6 months to identify those individuals at risk who would benefit from nutrition support (adjusted nutritional recommendations based on systemic symptom prevalence, oral supplements, tube feedings, and/or parenteral nutrition).

Low muscle mass is often not taken into consideration when interpreting and assessing energy needs and BMI. In PMD, the BMI z-score would likely be lower than the general population as it reflects genetically low muscle mass. Thus, PMD individuals with reduced muscle mass may be misinterpreted as having lower body weight. As BMI is a sole indicator in the pediatric AND/ASPEN criteria for malnutrition, there needs to be careful consideration in making a diagnosis of malnutrition in child PMD subjects. In a further example, an individual with a height on the 4th percentile and weight on the 17th percentile will yield a higher calculated BMI compared to an individual with a height on the 40th percentile. Hence, short stature in PMD may lead to misinterpretation of nutritional status if BMI is the sole indicator. Malnutrition assessments in PMD should thus be conducted by skilled RDNs to avoid errors in malnutrition diagnosis.

Studies have shown the impact of malnutrition on mitochondrial function. A hypocaloric diet consisting of 92 kJ/d for 7 days (n = 19) in rats led to a decrease of mitochondrial complex I and III activities (*p* < 0.01) after 10 days, in comparison to adult control rats fed 364 kJ/d for 10 days (n = 10) [85]. In a human study, Briet et al. studied malnourished adult patients (n = 15) and healthy volunteers (n = 30) admitted to a hospital [86]. One week of refeeding was followed by a significant improvement in the peripheral blood mononuclear cells (PBMC) complex I activity in malnourished patients, *p* < 0.02, which was as low as ~ 43% of complex I activity levels in healthy volunteers (*p* < 0.001); and that nutritional intake was directly proportional to the PBMC complex I activity level [86]. If chronic malnutrition occurs in PMD individuals, this likely aggravates the existing mitochondrial dysfunction related to PMD genetic etiology.

Macronutrient intake can be defined as absolute intake (g/d), intake related to body weight (g/kg/day), and intake as a fraction of total energy (% Kcal). Our cohort reportedly met a normal distribution of macronutrient consumption when based on the total energy consumed (Table e-9). However, when analyzed as a fraction of their WHO-MOTIVATOR predicted energy goal, relative reductions in overall macronutrient consumption became apparent (Table e-9). Subsequent assessment of absolute intake (g/day) (Table e-10) uncovered the observation of significantly low fat intake in comparison to population references [61, 87] across the PMD cohort. It is therefore important to also assess macronutrients in g/day as it may be better at discriminating macronutrient deficiencies. As DRI PRO and fat macronutrient goals (g/day) in children vary across the age range, interpretation of cohort mean values need to consider the age spectrum as there are considerable differences in predicted values between younger and older children.

In 28/60 (46.7%) PMD subjects, when their fat intake was low at < 30% of their total calories, it was observed that 11/28 (39.3%) subjects consumed > 60% of their daily calories as CHO, and 12/28 (42.9%) consumed > 20% of daily calories as PRO, suggesting a compensatory increase in CHO or PRO consumption. Whether this is due to PMD individual preference for CHO and PRO macronutrients on recognition that fat absorption is slower, which can intensify existing GI dysmotility symptoms, or is related to abnormal enteric cell mitochondrial fatty acid oxidation (FAO) affecting intestinal absorption; or from decreased systemic cellular capacity for FAO due to impaired nicotinamide adenine dehydrogenase balance that is common in PMD [88, 89] is not known. Fat is a major fuel substrate for endurance activities. The inadequate fat intake observed in our PMD cohort coupled to secondary mitochondrial fatty acid oxidation (FAO) impairment related to OXPHOS deficiency likely exacerbates muscle fatigue and exercise intolerance. Further, despite our PMD cohort reportedly meeting or exceeding their PRO needs, if subjects did not meet their overall energy goals, PRO intake would be diverted from its chief function of growth and repair to contribute to overall energy [55].

Several pre-clinical studies have demonstrated that modified macronutrient diets affect mitochondrial function. In rats, a low PRO diet (5% calories from PRO for 4 weeks) resulted in deformed and elongated mitochondria as well as reduced mitochondrial complex I and IV activity with downregulated pyruvate uptake [90]. In a separate study, a low PRO diet (8% casein for 15 days) resulted in reduced mitochondrial DNA content in rat liver and skeletal muscle tissue [91]. On the other hand, nephrectomized mice fed high PRO diets (30–50% calories from PRO for ~ 8 weeks) displayed increased muscle weight of the gastrocnemius, fiber size of the extensor digitorum longus, and urinary excretion of creatinine; however, muscle ETC enzymatic activity was decreased. This shows that a high PRO diet can reduce muscle mitochondrial function, despite increasing muscle volume [92]. In skeletal muscle of adult rats, body energy (kJ) and energetic efficiency (%) were found to be significantly higher after a 2-week high-fat/high-fructose diet, compared to a low-fat diet. Additionally, no significant differences in lipid peroxidation were observed [93]. In a small study (n = 4) of patients with mitochondrial complex I deficiency, the potential role of an isocaloric high-fat diet (triacylglycerol emulsion infusion of 20% intralipid) due to its ability to supply FADH_2_-linked reducing equivalents to the mitochondrial respiratory chain distal to complex I was studied. Isocaloric high-fat diet was associated with improved endurance, O_2_ consumption rates, and decreased plasma lactate levels following leg ergometry exercise as compared to measurements after a glucose infusion [94]. However, a more recent study reported that a high-fat low-carbohydrate diet can be acutely detrimental in PMD subjects, where a modified ketogenic diet resulted in increased muscle pain and muscle damage as well as lysis of ragged-red fibers (RRFs) [95]. Given results of significantly decreased fat intake in our PMD cohort, we anticipate that the optimal and likely more tolerable approach in a future clinical trial would be the optimization of macronutrient and caloric intake, as opposed to a high-fat macronutrient diet.

Limitations of this study include: (1) Reliance on the accuracy of PMD subject recall. Only a small subset of subjects completed the 3 day-diet records ahead of their RND clinic visit. As documentation of diet records is not currently routine practice in our center’s Mitochondrial Medicine clinic, PMD patient education on how to complete 3-day diet record documentation and its clinical relevance will be needed. (2) An assumption that the WHO-REE predictive equation is reliable in PMD. Further study comparing the WHO-REE to IC is required for confirmation. (3) Weight and height measurements were obtained in our Mitochondrial Medicine clinic by the medical assistants (MAs) and verified by the RDN. Although MAs are trained at our institution and used the same equipment across the cohort, the accuracy of clinic measurements is less optimal when compared to measurements obtained as a research procedure, as has been shown in other studies [80–82]. Further, weight and height measurements obtained across different clinics using varying equipment and measurement methods contributes to the observed variability. (4) Objective myopathy measures and survey assessments were not completed by the full study cohort. In the future, a systematic study of nutrition involving these assessments in the study protocol is needed.

In summary, our study highlights the importance of regularly obtaining detailed nutritional assessment in PMD subjects to evaluate their ability to meet total daily energy and macronutrient intake goals in the context of growth, PMD symptoms, functional capability, and quality of life. In our systematic approach, we proposed the need to implement a customized AF (MOTIVATOR) assessment tool to predict TEE in PMD subjects. We emphasized the need to complement the conventional approach of expressing PMD macronutrient intake as a percentage of consumed energy, with expression as a percent of predicted energy intake, and as g/day. Importantly, we identified significant correlations of increased macronutrient and Kcal intake in a subset of PMD subjects to objective measurement of muscle fatigue, strength, and QoL scores. Systematic study would be critical to identify tolerable and effective nutritional modulation strategies to be tested in a future clinical trial of PMD subjects, where our results would also provide the foundation to inform an optimal clinical trial design. The MM-COAST measures and surveys utilized in this study could be considered to provide clinically meaningful end-points to objectively measure how a patient functions and feels in response to a nutritional intervention.

### Supplementary Information

Below is the link to the electronic supplementary material.

Supplementary file1 (PDF 866 KB)

## Data Availability

All deidentified data supporting the findings of this study are available within the paper and its Supplementary Information. Additionally, deidentified data are available from the corresponding author upon reasonable request and with permission from The Children’s Hospital of Philadelphia Institutional Review Board.
