# Supplementary material for: Optimized Nutrition in Mitochondrial Disease Correlates to Improved Muscle Fatigue, Strength, and Quality of Life
Source: Neurotherapeutics. 2023 Sep 18;20(6):1723–45. doi: 10.1007/s13311-023-01418-9 (PMC10684455; doi:10.1007/s13311-023-01418-9)
Supplement: Supplementary file 1 — Supplementary file1 (PDF 866 KB) [file 13311_2023_1418_MOESM1_ESM.pdf]

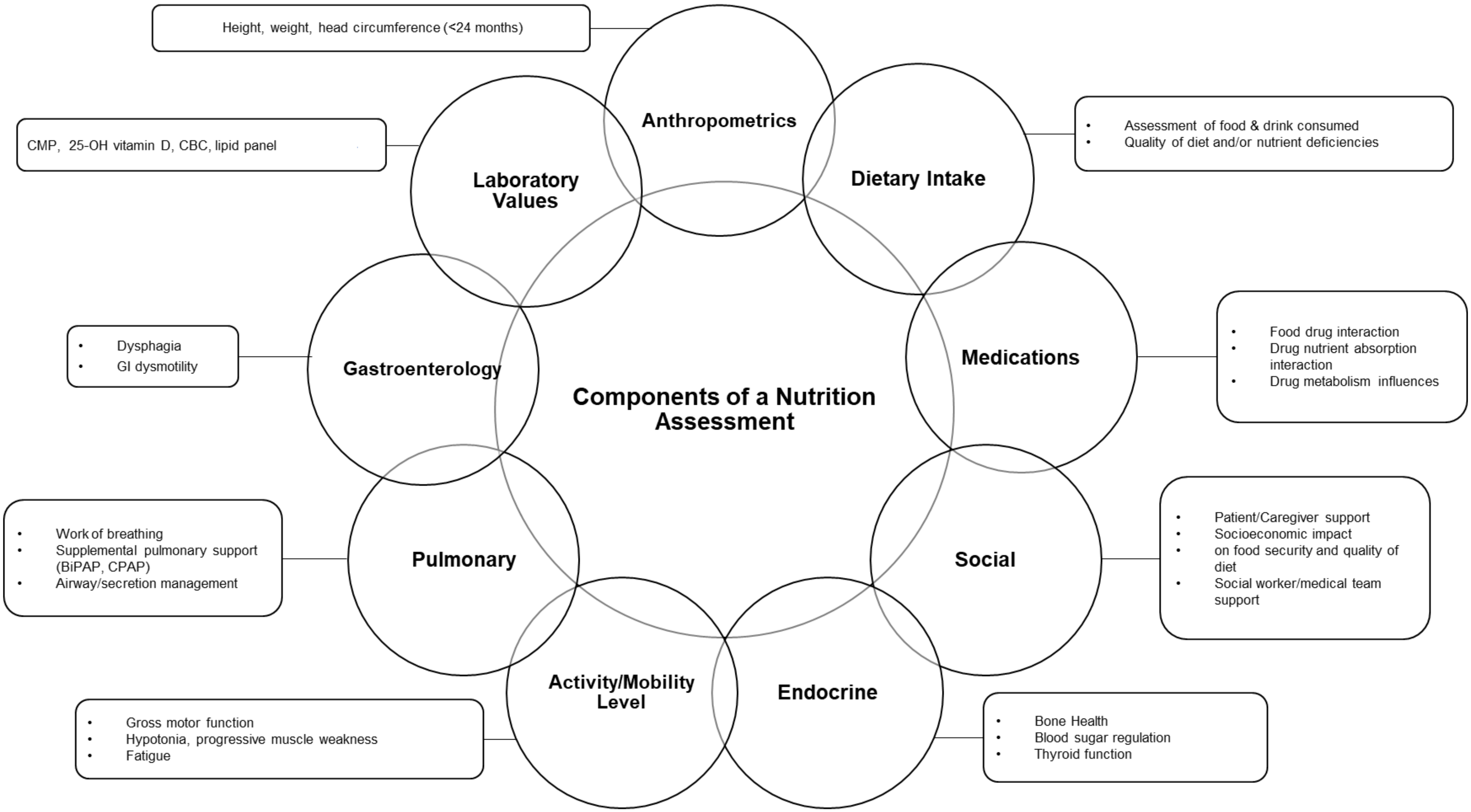

Supplemental Fig. 1 Components of a Nutrition Assessment. Supplemental Figure 1 highlights the 9 major components of a detailed nutritional evaluation that should include assessments of medications, muscle mass and strength, fatigue, bone health, and dysphagia to ensure that that all relevant aspects of the medical history are carefully considered in the assessment.

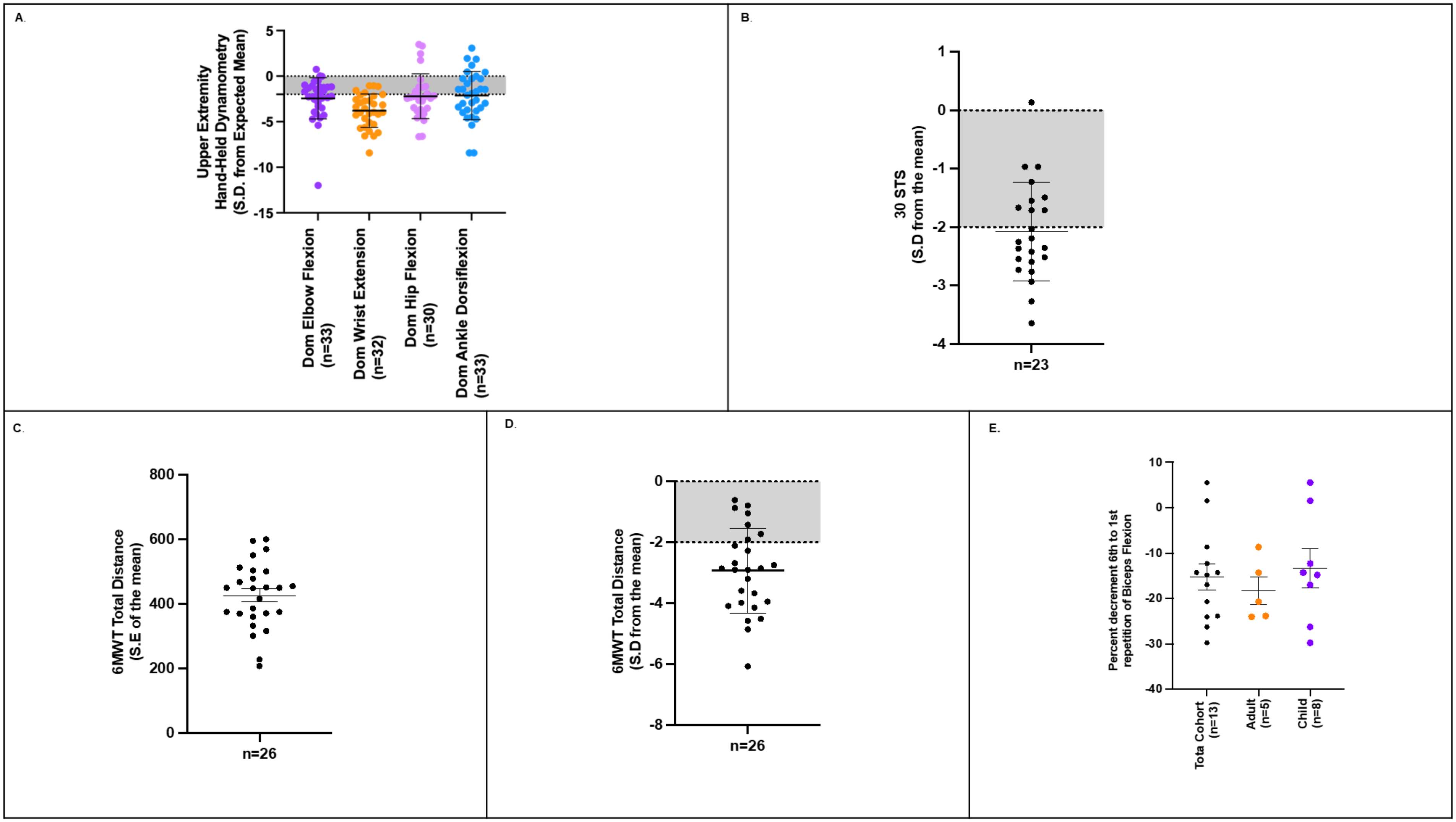

Supplemental Fig. 2

(A) Upper and Lower Extremity Hand-Held Dynamometry z-scores. Results are presented as mean ± standard deviation (S.D., where z-score ≤ -2 is considered abnormal. Mean z-scores for upper extremity measurements revealed wrist extension strength as -3.8 ± 1.9, (n=32) and elbow flexion -2.5 ± 2.2, (n=

33). In the lower extremities, mean hip flexion z-score was -2.2 ± 2.5 (n=30), and ankle dorsiflexion was -2.1 ± 2.7 (n=33). Overall, dynamometry measurements revealed muscle weakness in all muscle groups.

(B) 30s Sit-to-Stand (30s STS) Exercise Intolerance Test. In terms of exercise intolerance, the mean z-score for 30s STS was -2.01 ± 0.84 (n=23).

(C) 6-Minute Walk Test (6MWT) Total Distance. Mean total distance walked for 6MWT was 425.6 ± 20.1 (n=26), mean ± SEM (meters).

(D) 6MWT Mean z-score. The mean total distance walked for 6MWT corresponds to a mean Z-score of -2.9 ± 1.4 (n=26), mean ± SD [98].

(E) Repeated Hand-Held Dynamometry Muscle Fatigue Assessment. The negative percent decrement for dominant elbow flexion was -15.3 ± 2.9% (SEM, n=13) [adults, -18.3 ± 3.0%, n=5; child, -13.4 ± 4.3%, n=8]. In a small cohort of healthy volunteers published elsewhere [28], dominant elbow flexion negative percent decrement was documented to be lower at -8.3 ± 2.4% (n=29) [adult, -6.5 ± 4.4%, n=15; child, -10.1 ± 1.9%, n=14].

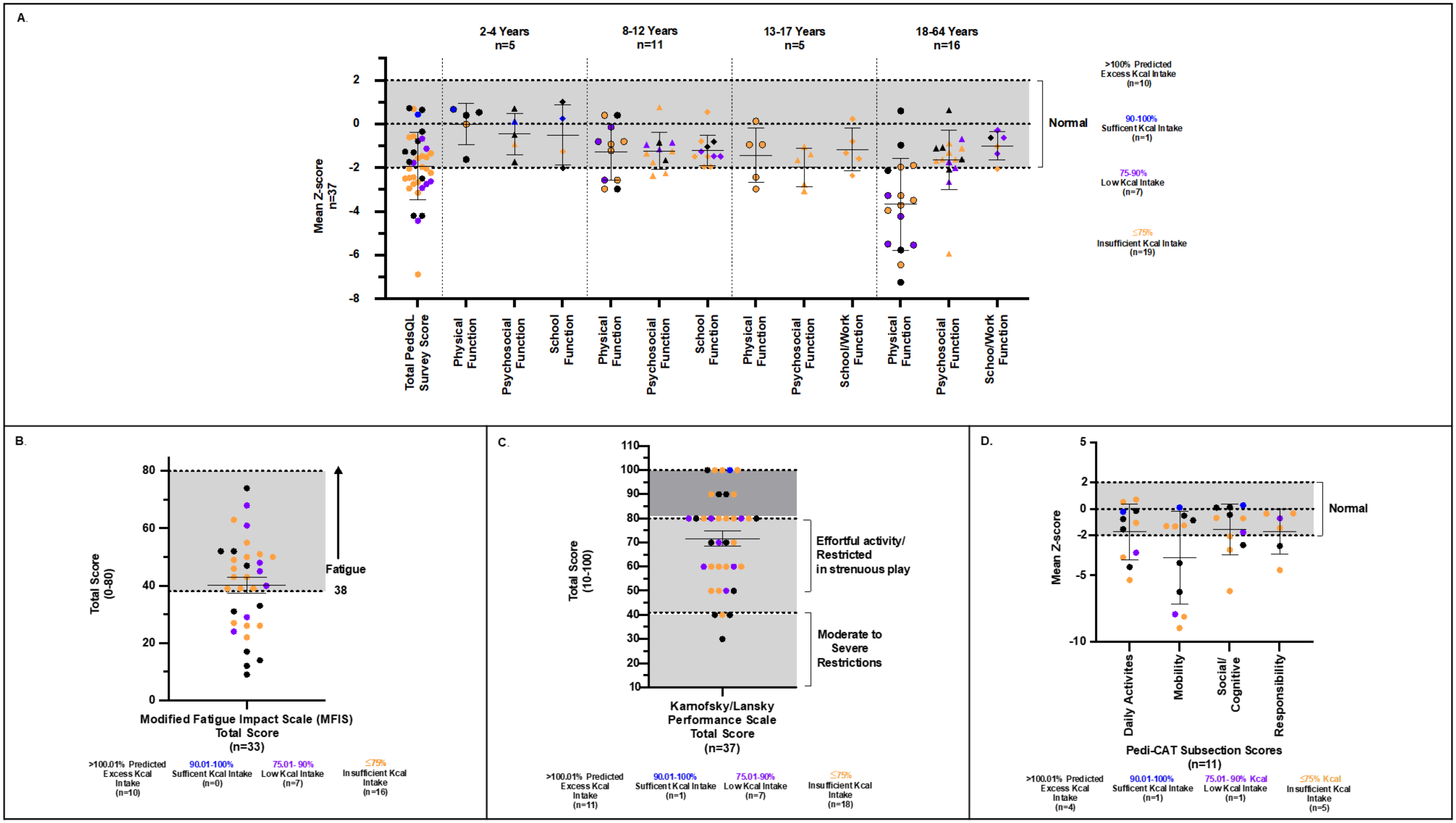

Supplemental Fig. 3

(A) Pediatric Quality of Life Survey mean z-scores by Age groups and by subsections (n=37). PedsQL consists of a total score, and 3 subsection scores, the physical function, psychosocial and school/work function (age dependent). Cohort mean overall z-score was -1.9 ± 1.5. Subjects are sorted into 4 age groups, 2-4 years (n=5), 5-7 yeas, 8-12 years (n=11), 13-17 years (n=5), and 18+ years (n=16). In the 2–4 year-old group)n=5),

mean physical, psychosocial and school function z-scores were -0.01 ± 0.9, -0.4 ± 0.9, -0.5 ± 1.4, respectively. In the 8-12 year-old group (n=11), mean z-scores were -1.3 ± 1.3 for physical function, -1.2 ± 0.9 for psychosocial function and -1.2 ± 0.7 for school function. In the 13-17 year old group (n=5), z-scores were -1.4 ± 1.3 for the physical function subsection, -1.98 ± 0.9 for psychosocial function and for school/work function, was -1.4 ± 0.9. In the 18+ year group (n=16), scores were -3.7 ± 2.1 for physical function, -1.7 ± 1.4 for psychosocial function, and -1.0 ± -0.6 for school/work function.

(B) Modified Fatigue Impact Scale (MFIS) (n=33). Mean total score for the PMD cohort was 40.1 ± 2.8, consistent with fatigue.

(C) Karnofsky/Lansky Performance Scale (n=37). Cohort mean total score was 71.6 ± 3.2, consistent with effortful activity/restricted in strenuous play.

(D) Pedi-CAT Subsection Scores (n=11). Z-scores are calculated for Daily Activities, Mobility, Social/Cognitive, and Responsibility subsections, with normative z-score of 0 ± 2. The normal range is ± 2SD. Z-scores of -1.7 ± 2.1 in daily activities, -3.7 ± 3.5 in mobility, -1.6 ± 1.9 in social/cognitive, and -1.7 ± 1.7 in responsibility (n=6), indicated low performance and skill in mobility.

**Table e-1. Primary Mitochondrial Disease Genetic Etiologies**		
**Nuclear DNA Etiologies<br>(n=25)**		
**Genes**	**Number (%)**	**Pathogenic or Likely Pathogenic Variants**
POLG	7 (28)	Individual 1: c.752C>T:p.T251I + c.1760C>T:p.P587L (in *cis*) and c.1880G>A:p.R627Q[^]<br>Individual 2: c.2669A>C:p.D890A<br>Individual 3: c.2209G>C:p.G737R and c.926G>A:p.R309H[^]<br>Individual 4: c.32G>A:p.G11D + c.2554C>T:p.R852C (in cis), and c.2243G>C:p.W748S[^]<br>Individual 5: c.2209G>C:p.G737R and c.1399G>A:p.A467T[^]<br>Individual 6: c.202C>T:p.Q68* and c.911T>G:p.L304R[&]<br>Individual 7: c.1399G>A:p.A467T and c.2243G>C:p.W748S[^]
COQ8A	3 (12)	Individual 1: c.811C>T:p.R271C and c.655+3A>C[^]<br>Individual 2: c.1042C>T:p.R348X and c.830T>C:p.L277P[^]<br>Individual 3: c.1042C>T:p.R348X and c.830T>C:p.L277P[^]
SURF1	2 (8.0)	Individual 1: c.751C>T:p.Q251X and c. 532_535delAATA:p.N178EfsX9[^]<br>Individual 2: c.312_321del10insAT:p.L105X and c.269T>C:p.L90P[^]
AFG3L2	2 (8.0)	Individual 1: c.1153G>A:p.G385S[#]<br>Individual 2: c.571G>A:p.V191I[&]
TRMU	2 (8.0)	c.1084G>A:p.A362T[^] and partial gene deletion
TWNK	1 (4.0)	c.1110C>G: p.F370L[&]
MPV17	1 (4.0)	c.191C>T:p.P64L and c.375+5G>T[^]
DLD	1 (4.0)	c.1123G>A:p.E375K (confirmed homozygous)
MRPS34	1 (4.0)	c.407G>C:p.R136P and c.200dupT:p.L68Afs*65[^]
NUBPL	1 (4.0)	c.166G>A:p.G56R + c.815-27T>C:IVS9-27T>C (in cis), and c.693+1G>A:IVS8+1G>A[^]
PDHA1	1 (4.0)	c.409G>A:p.E137K
WARS2	1 (4.0)	c.37T>G:p.W13G and c.683C>G: p.S228W[^]
ACAD9	1 (4.0)	c.1237G>A:p.E413K and c.1552C>T:p.R518C[^]
SSBP1	1 (4.0)	c.79G>A:p.E27K
[#]*de novo*; [^]confirmed *in trans;* [&]parental samples unavailable		
**Mitochondrial DNA Etiologies<br>(n=35)**		
**Genes**	**Number (%)**	**Pathogenic or Likely Pathogenic Variants[*]**
Single large-scale deletions	10 (29)	a. 50%[M]<br>b. 58%[Bl]<br>c. 30%[Bl]<br>d. 23%[Bl]<br>e. 15%[Bl]<br>f. Heteroplasmy levels not measured (n= 5)

MT-TL1	4 (11)	m.3243A>G<br>a. 67%^{M}<br>b. 25-50%^{Buc}<br>c. 45%^{Buc}<br>d. 22%^{Buc}, 12%^{Bl}
MT-ATP6	3 (9.0)	m.9185T>C<br>a. 98%^{Bl}<br>b. 95%^{Bl} (with co-existent m.15243G>A 5%^{Bl})<br>c. Heteroplasmy levels not measured (n=1)
	2 (6.0)	m.8993T>G<br>a. 97%^{Bl}<br>b. Homoplasmic
MT-ND3	3 (9.0)	m.10191T>C<br>a. 83%^{Buc}, 87%^{U}, 84%^{Bl}<br>b. 78%^{Buc}, 44%^{Bl}<br>c. 76%^{Bl}
	1 (3.0)	m.10197G>A<br>a. 90%^{Bl}
	1(3.0)	m.10158T>C<br>a. 77%^{Bl}
MT-TK	4 (11)	m.8344A>G<br>a. 75%^{Buc}<br>b. 88%^{Bl}<br>c. 88%^{Bl}<br>d. 80%^{Bl}
MT-ND5	3 (9.0)	m.13513G>A<br>a. 80%^{U}, 48%^{Bl}<br>b. 67%^{U}, 22%^{Bl}<br>c. 69%^{Bl}
MT-ND1	2 (6.0)	m.3460G>A<br>a. 89%^{U}, 47%^{Bl}<br>b. Heteroplasmy levels not measured (n=1)
MT-TF	1 (3.0)	m.636A>G<br>a. 56%^{Buc}, 63%^{Bl}
MT-ND6	1(3.0)	m.14459G>A<br>a. 88%^{Bl}

*Heteroplasmy levels in various tissues, listed by individual subjects (a-f): Bl = peripheral blood; Buc = Buccal tissue; Sal = Saliva; M = Skeletal Muscle; U = Urine sediment

Table e-2. Primary Mitochondrial Disease (PMD) subjects BMI classification [1, 2]				
**Number (%)**	**PMD Adult* (n=22)**	**PMD Adult with G-tube (n=2/22)**	**PMD Child^ (n=38)**	**PMD Child with G-tube (n=6/38)**
**Underweight**	3 (13.6%)	-	12 (31.6%)	4 (66.7%)
**Appropriate**	13 (59.1%)	1 (50%)	20 (52.6%)	1 (16.7%)
**Overweight/Obese**	6 (27.3%)	1 (50%)	6 (15.8%)	1 (16.7%)

*Adult BMI classification for adults according to ASPEN classification: Underweight (BMI 18.5), Appropriate (BMI 18.5-25), Overweight (BMI 25-29.9), and Obese (BMI 30 and above) [1]

^BMI scores < 5th percentile were classified as Underweight, 5th-84th percentile were Appropriate, 85th-97th percentile were Overweight, and > 97th percentile were Obese

Abbreviation(s): BMI - Body Mass Index

Table e-3. Patient-reported GI Symptoms in Primary Mitochondrial Disease (PMD)					
	PMD Cohort (n=60)	Adult Cohort (n=22)	Child Cohort (n=38)	All PMD subjects who consumed <75% predicted Kcal (n=29)	All PMD subjects who consumed 75.01 -100% predicted Kcal (n=14)
**Dysphagia**	28 (46.7%)	13(59.1%)	15 (39.5%)	12 (41.4%)	6 (42.9%)
**Constipation**	26 (43.3%)	11 (50.0%)	15 (39.5%)	15 (51.7%)	5 (35.7%)
**Nausea**	7 (11.7%)	5 (22.7%)	2 (5.3%)	3 (10.3%)	4 (28.6%)
**Reflux**	14 (23.3%)	7 (31.8%)	7 (18.4%)	6 (20.7%)	6 (42.9%)
**Vomiting**	14 (23.3%)	4 (18.2%)	10 (26.3%)	9 (31.0%)	3 (21.4%)
**GI dysmotility (slow gastric emptying)**	15 (25.0%)	8 (36.4%)	7 (18.4%)	8 (27.6%)	5 (35.7%)
**Abdominal pain**	6 (10.0%)	3 (13.6%)	3 (7.9%)	2 (6.9%)	3 (21.4%)
**Diarrhea**	10 (16.7%)	6 (27.3%)	4 (10.5%)	5 (17.2%)	4 (28.6%)
**Loss of appetite**	7 (11.7%)	2 (9.1%)	5 (13.2%)	5 (17.2%)	1 (7.1%)
**Poor weight gain**	7 (11.7%)	2 (9.1%)	5 (13.2%)	3 (10.3%)	1 (7.1%)
**Difficulty feeding**	6 (10.0%)	0 (0%)	6 (15.8%)	2 (6.9%)	1 (7.1%)
**Failure to thrive**	12 (20.0%)	4 (18.2%)	8 (21.1%)	4 (13.8%)	1 (7.1%)

**Table e-4. Comparison of Daily Kcal Intake using Conventional World Health Organization [3] (WHO) vs. Harris Benedict (HB) [4] vs Mifflin-St. Jeor [5] Resting Energy Expenditure Equations in adult subjects***				
**Mean ± SEM (Kcal/day, range)**	**Adult (n=22)**			**ANOVA *p-value*#**
	**WHO**	**HB**	**MSJ**	
**Predicted Kcal Intake***	1,936 ± 59.0 (1,417 – 2,397)	1,938 ± 50.1 (1,587 – 2,346)	1,827 ± 62.5 (1,324 – 2,276)	0.30
**Estimated daily Kcal intake**	1,143 ± 104.1 (226- 2,340)			-
**Percent predicted Kcal intake (%)**	60.1%	59.5%	63.8%	0.84
**T-test *p-value*^**	<0.0001	<0.0001	<0.0001	

*Predicted Kcal intake calculation: Resting Energy Expenditure value x ASPEN/RDA Activity Factor

^Comparison by paired parametric t-test of Predicted Kcal intake vs. Estimated daily Kcal intake: significant difference is $p<0.05$

#One-way ANOVA comparison of predicted Kcal intake and percent (%) predicted Kcal intake using WHO vs HB vs MSJ equations

Abbreviation(s): ASPEN - American Society for Parenteral and Enteral Nutrition, RDA - Recommended Daily Allowance, WHO - World Health Organization, HB - Harris Benedict, MSJ - Mifflin-St. Jeor

Table e-5. Comparison of Daily Kcal Intake using ASPEN/RDA [1, 6] Activity Factors and Primary Mitochondrial Disease (PMD)-specific Activity Factors (MOTIVATOR)*						
**Mean ± SEM (Kcal/day, range)**	**Adult (n=22)**		**T-test *p-value***	**Child (n=38)**		**T-test *p-value*#**
	**ASPEN**	**MOTIVATOR**		**ASPEN**	**MOTIVATOR**	
**Predicted Kcal Intake***	1,936 ± 59.0 (1,417 – 2,397)	1,554 ± 58.0 (1,012 -2,058)	<0.0001	1,633 ± 94.5 (535.6 - 3,195)	1,444 ± 84.9 (469.8 – 2,631)	<0.0001
**Estimated daily Kcal intake***	1,143 ± 104.1 (226- 2,340)		-	1,114 ± 62.3 (410 – 2,314)		-
**Percent predicted Kcal intake (%)**	60.1%	76.2%	<0.0001	76.5%	86.4%	<0.0001
**T-test *p-value*^**	<0.0001	0.003	-	<0.0001	0.001	-

*Predicted Kcal intake calculation: WHO Resting Energy Expenditure value x Activity Factor

^Comparison by paired-parametric t-test between Predicted Kcal intake vs. Estimated daily Kcal intake

#Comparison by paired-parametric t-test between Percent predicted Kcal intake using ASPEN/RDA vs. Mitochondrial Disease specific Activity Factors

Abbreviation(s): ASPEN - American Society for Parenteral and Enteral Nutrition, RDA - Recommended Daily Allowance, WHO - World Health Organization

Table e- 6. Mitochondrial Activity Factors (MOTIVATOR)

Instructions for Estimation of Total Energy Needs in Individuals with Primary Mitochondrial Disease:*
Total Calculated Energy Needs= Resting Energy Expenditure [3]** x **Activity Factor (AF) ± Modification**

1) Select **Activity Factor** based on corresponding activity level, motor/mobility function, and fatigue characterization (must meet at least one criterion in 'Motor/Mobility Function and Fatigue characterization' column).
2) **Modify** Activity Factor using the following considerations:
    a. **Increased Energy Demand:** Add a range of 0.1-0.3 to the selected AF based on clinical judgement if any of the following are present:
        - Uncontrolled extrapyramidal movements such as spasticity, chorea, dystonia, significant ataxia impacting gait or other abnormal movements
    b. **Decreased Energy Demand:** Subtract a range of 0.1-0.3 from the selected AF based on clinical judgement if any of following are present:
        - Muscle hypotonia
        - Decreased muscle mass
        - Mechanical ventilation
        - Medications (muscle relaxants - example Baclofen and anti-seizure medications)
        - Excessive daytime sleep leading to prolonged sedentary periods
    c. **Fatigue:**
        - If an individual meets the criteria for an activity level, however, needs a recovery/rest period 1-2 days following that amount of activity, score down one activity level **and** apply the highest AF from the stated range of that level

***Clinical judgement should be utilized to assess if further adjustments in estimated energy intake need to be considered based on patterns of growth, body composition, medications, and acute illness.
****Resting Energy Expenditure **(**REE) to be calculated using indirect calorimetry, or prediction equation such as the WHO or Harris Benedict

©2020 The Children's Hospital of Philadelphia. All rights reserved.

Table e-7. Pediatric (age 1 through 18 years) Mitochondrial Activity Factors (MOTIVATOR)			
**AF**	**Activity Level**	**Motor Function and Fatigue Characterization**	**Description**
**1**	Level 1:<br>Minimal	• Unable to walk<br>• Dependent for transfers<br>• Requires support for head, neck, and trunk position for seating<br>• Limited active movement of limbs<br><br>Specific Motor Activities:<br>• Walking<br>○ Needs to be pushed in a fully supportive adapted stroller or wheelchair (head and trunk supports, tilt-in-space, or reclined).<br>• Stair Climbing<br>○ Unable to climb stairs or use stair glide. Carried by caregiver or stairs avoided.<br>• Swinging at Playground<br>○ Parent/caregiver places in fully supportive adapted swing and parent/caregiver pushes swing.<br>• Swimming<br>○ Requires fully supportive adapted swimming flotation devices and full assistance by parent/caregiver, minimal movement of extremities in water.	Transported in manual wheelchair or adapted stroller
**1.1-1.3**	Level 2:<br>Very Light	• Unable to walk<br>• Sits independently but not able to move into or out of this position<br>• Rolling, or crawling on belly, or creeping on all fours less than 10 ft<br>• Physical assistance required for transfers or transitions<br>• Able to weight bear, may take 5-10 steps with physical assistance or support with walker or gait trainer<br>• Use of wheelchair or adapted stroller<br>• May drive power wheelchair<br><br>Specific Motor Activities:<br>• Walking<br>○ May drive power wheelchair or be pushed in manual wheelchair. Able to sit upright and move extremities<br>• Stair Climbing	Short bursts of activity on the floor, rolling or crawling, sits to play with toy briefly, sits in supportive chair to play with toy, color, or paint, or use iPad.

		○ Unable to climb stairs. Able to sit in a stair glide chair to be transported up steps<br>• Swinging at Playground<br>○ Parent/caregiver places in age-appropriate swing, able to hold onto swing and kick legs but unable to “pump” swing, needs to be pushed by parent/caregiver<br>• Swimming<br>○ Requires a flotation device or support of parent/caregiver but can kick and move arms and legs in the water, but does not propel across water, remains in place (treading water), may stand without support in chest deep water and take steps with hand-held assist or holding edge of pool	
**1.3-1.4**	Level 3: Light	• Walks independently using assistive device (walker, crutches, gait trainer) or hand-held assistance from caregiver, or short distances unsupported indoors<br>• When seated, hands are free to play<br>• Able to move into and out of sitting independently<br>• Crawls independently around room<br>• Cruises along furniture<br>• Some physical assistance required for difficult transfers such as getting up off the floor<br>• Physical assistance required for stair climbing<br>• Requires use of wheelchair, stroller, or scooter for community level distances and requires adapted equipment for sports<br><br>Specific Motor Activities:<br>• Walking<br>○ Walks a short distance indoors using an assistive device (walker, crutches, gait trainer) or hand-held assistance from caregiver or <10 independent steps with supervision. May propel manual wheelchair short distances indoors<br>• Stair Climbing<br>○ Parent or caregiver assistance required for stair climbing.<br>○ Younger children:<br>▪ Crawls up a few steps but <1 flight<br>• Swinging at Playground	Prefers sedentary activities such as sitting to play, video games, reading, painting and drawing, doing puzzles, crawls or walks short distances with a walker or across room to get to sitting activity, may ride adapted bike with assistance

<table>
<tr><td></td><td></td><td><li style="list-style:none">
Parent places in age-appropriate swing, able to hold onto swing and move trunk and leg in pumping motion but unable to start swing without assistance, able to maintain balance and sustain swinging, while being pushed by parents intermittently for at least 5 minutes
Swimming
May need minimal flotation device such as a tube, “swimmies” (inflatable arm bands), a pool noodle, kick board, or swim vest, but able to kick and propel across short distances (10 ft or less), may be able to stand without support and take steps in chest deep water at least 10 steps without support. In general plays in small area of pool</td><td></td></tr>
<tr><td>1.4-1.5</td><td>Level 4: Moderate with Fatigue</td><td>Able to stand and walk independently indoors and outdoors including home and school
No assistive devices needed/may wear orthotics
Limited by fatigue:
Able to walk 1/4 mile (5 city blocks) but requires rests
Able to walk up one full flight but not 2 flights of steps without rest
Able to run < 1 minute before requiring rest
Able to sit independently and play with arms free but requires rest or leans on support in < 10 minutes.
<u>Younger children (developmental age 1-2 years):</u>
Able to transition in and out of sitting, crawling, and pulls to stand independently
Able to crawl throughout house, requires rest after ≤ 10 minutes of activity
Specific Motor Activities:
Stair Climbing
Able to walk up one full flight but not 2 flights of steps without rest. No assistance needed
Younger children:
Able to crawl up 1 flight of steps but not 2 flights independently without resting
Swinging at Playground</td><td>Can participate in the following but requires rests throughout: walks, plays at playground, horseback riding, bike riding, recreational swimming</td></tr>
</table>

		○ Child attains sitting on swing independently (younger child may be assisted by parent/caregiver), able to pump independently without assistance, and sustain swinging for <5 minutes before needing a rest.<br>• Swimming<br>○ Able to enter and exit pool independently (age-appropriate assistance for younger toddler/child), able to move independently walking in chest deep water or swimming with minimal (age appropriate) or no flotation device. Able to propel him/herself across the pool at least 20 ft. Able to sustain activity in the water <=10 minutes before requires rest.	
**1.5-1.7**	Level 5: Moderate without Fatigue	• Able to stand and walk independently indoors and outdoors including home and school<br>• No assistive devices needed/may wear orthotics<br><br>• Able to walk 1/4 mile (5 city blocks) without rest<br>• Able to walk up 2 flights of steps without rest<br>• Able to run > 1 minute without rest<br>• Able to sit independently and play with arms free without leaning on support >=10 minutes<br><br><u>Younger children (developmental age 1-2 years):</u><br>• Able to transition in and out of sitting, crawling, and pulls to stand independently<br>• Able to crawl reciprocally throughout house > 10 minutes without rests<br><br>Specific Motor Activities:<br>• Stair Climbing<br>○ Able to walk up 2 flights of steps without rest. No assistance needed<br>○ Younger children:<br>▪ Able to crawl up 2 flights of steps independently without resting<br><br>• Swinging at Playground<br>○ Child attains sitting on swing independently (younger child may be assisted by parent/caregiver), able to pump independently without assistance, and sustain swinging for 5-10 minutes before needing a rest.<br>• Swimming	Walks, plays at playground, horseback riding, bike riding, recreational swimming.

		○ Able to enter and exit pool independently (age-appropriate assistance for younger toddler/child), able to move independently walking in chest deep water or swimming with minimal (age appropriate) or no flotation device. Able to propel him/herself across the pool at least 20 ft. Able to sustain activity in the water 11-29 minutes before requires rest.	
**1.7-2**	Level 6: Heavy without limitations	• Able to walk, run, play, and participate in activities or sports independently without limitations. Able to run and jump and walk on uneven terrain. Keeps up with peers.<br><br>Specific Motor Activities:<br>• Walking<br>○ Able to walk independently throughout community on all surfaces and uneven terrain without limitations. May wear orthotics. Able to keep up with peers, no rests needed.<br>• Stair Climbing<br>○ Able to walk up multiple (>2) flights of steps at home, in school, and in the community without any limitations or rests needed<br>• Swinging at Playground<br>○ Child attains sitting on swing independently (younger child may be assisted by parent/caregiver age appropriately), able to pump independently without assistance and sustain swinging >10 minutes.<br>• Swimming<br>○ Able to enter and exit pool independently (age-appropriate assistance for younger toddler/child), able to move independently walking in chest deep water or swimming with minimal (age appropriate) or no flotation device. Able to propel him/herself across full length of standard sized pool (25 yards/75 feet) and swim laps without flotation device. Able to sustain activity in the water >30 minutes without rest. Able to keep up with peers.	Participates in gym class without adaptation, may participate in sports (on school team or recreational). Other activities may include: soccer, basketball, hiking, running, dance class, gymnastics, and/or karate.

Table e-8. Adult (age ≥ 19 years) Mitochondrial Activity Factors MOTIVATOR			
**AF**	**Activity Level**	**Motor Function and Fatigue Characterization**	**Description**
**0.8-1.0**	Level 1:<br>Minimal	• Unable to walk<br>• Requires support for head, neck, and trunk position for seating<br>• Dependent assist required for transfers<br>• Limited movement of limbs<br>• Use of wheelchair (pushed in manual or may drive power)	Transported in a manual wheelchair or drives power wheelchair. Activates switches with finger movements or eye gaze.
**1.0-1.1**	Level 2:<br>Very Light	• Some assistance required for transfers<br>• Seated activities or limited (≤5 minutes) standing activities<br>• Use of wheelchair (manual or power)<br>• May walk short distances in home but requires physical assistance and use of assistive devices/walkers	Activities may include: painting, writing, driving, playing cards, playing video games, sewing or knitting, self-feeding, dressing self in the seated position
**1.1-1.2**	Level 3:<br>Light	• Walks independently in the home-may use assistive device<br>• Walks more slowly than peers<br>• Able to stand for activities at least 10 minutes before requiring rest<br>• Community level mobility requires use of walker, wheelchair, or scooter outside the home or requires frequent rests (able to walk one block or less before needs to rest)<br>• Able to walk up one flight of steps but does so slowly or requires rest	Difficulty keeping up with peers, may play piano or guitar, wash dishes, completes most self-care independently including showering/ feeding/ dressing etc.<br><br>Spends 75% of time sitting or standing still, 25% of time standing and moving throughout the day
**1.2-1.3**	Level 4:<br>Moderate with Fatigue	• Able to walk independently in home and on even surfaces outdoors without walking aides /may wear orthotics<br>• Able to walk 1 mile but requires rest<br>• Able to walk up one flight but not 2 flights of steps without rest. No assistance needed<br>• Able to participate in activities (see examples) independently but requires frequent rests (within 10 minutes or less)	Activities (requires rest after 10 minutes or less):<br>Gardening, vacuuming, cycling (leisurely), shopping, carrying groceries into house, walking a dog
**1.3-1.4**	Level 5:<br>Moderate without Fatigue	• Able to walk independently in home and on even surfaces outdoors without walking aides /may wear orthotics<br>• Able to walk 1 mile without rest<br>• Able to walk up 2 flights of steps independently without rest	Activities (>10 minutes without rest):<br>Gardening, vacuuming, cycling (leisurely), shopping,

		• Able to participates in activities (see examples) for >10 minutes independently without requiring rests	carrying groceries into house, walking a dog
**1.4-1.5**	Level 6: Heavy	• Able to walk on all surfaces (even and uneven/elevations)<br>• Able to run and participate in sports independently<br>• Able to walk on all surfaces, including up and down hills >1 mile without difficulty or rest<br>• Stair climbing: able to walk up ≥3 flights of steps without use of railing and without resting<br>• Able to participate in activities (see examples) without difficulty or rests	Activities (without difficulty or rest):<br>Hiking, basketball, soccer, tennis, strength training at gym, dancing, regular exercise at least 3x/week

©2020 The Children's Hospital of Philadelphia. All rights reserved.

Table e-9. Comparison of Macronutrient Consumption to the Recommended Daily Allowance (RDA) [6, 7]						
**Mean ± SEM**	**Adult (n=22)**			**Child (n=38)**		
	**Carbohydrate (CHO)**	**Protein (PRO)**	**FAT**	**CHO**	**PRO**	**FAT**
**Estimated macronutrient consumption (Kcal/day)**	627.6 ± 81.0	219.2 ±18.5	306.1 ± 29.6	571.1 ± 40.7	190.0 ± 12.8	365.1 ± 23.4
**Estimated daily Kcal intake (Kcal/day)****	1,143 ± 104.1			1,114 ± 62.3		
**Mean macronutrient consumption percentage of Estimated Kcal intake (%)[a]**	52.9 ± 2.2%	20.2 ± 1.6%	27.4 ± 1.5%	50.1 ± 1.7%	17.5 ± 1.1%	33.5 ± 1.4%
**RDA predicted macronutrient distribution goal (mid-point, AMDR*)**	55 (45-65%)	15 (10-35%)	30 (20-35%)	50 (45-65%)	20 (15-30%)	30 (25-35%)
**Percent predicted when compared to macronutrient RDA goal (%)****	96.2%	134.7%	91.5%	101.3%	87.5%	109.7%
**Estimated macronutrient consumption (Kcal/day)****	627.6 ± 81.0	219.2 ±18.5	306.1 ± 29.6	571.1 ± 40.7	190.0 ± 12.8	365.1 ± 23.4
**WHO-MOTIVATOR predicted Kcal intake (Kcal/day)**	1,554 ± 58.0			1,444 ± 84.9		
**Mean macronutrient consumption percentage of WHO-MOTIVATOR predicted Kcal intake (%)[b]**	42.1%	14.5%	20.5%	44.1%	14.3%	29.0%
**RDA predicted macronutrient distribution goal (mid-point, AMDR*)**	55 (45-65%)	15 (10-35%)	30 (20-35%)	50 (45-65%)	20 (15-30%)	30 (25-35%)
**WHO- MOTIVATOR predicted macronutrient goal based on RDA distribution (Kcal/day)**	849.2 ± 31.9	231.5 ± 8.7	463.1 ± 17.4	722.2 ± 42.5	285.5 ± 17.9	436.6 ± 24.7
**Percent predicted when compared to macronutrient RDA goal (%)[c]**	76.5%	96.8%	68.2%	89.7%	71.7%	94.2%

[a] Equation: Estimated macronutrient consumption (Kcal/day)/ RDN Estimated Kcal intake (Kcal/day)

[b]Equation: Estimated macronutrient consumption (Kcal/day)/ WHO-MOTIVATOR predicted Kcal intake (Kcal/day)
[c]Equation: [Estimated macronutrient consumption (Kcal/day)/ WHO-MOTIVATOR predicted Kcal intake (Kcal/day)]/ RDA predicted macronutrient distribution goal (%)
*AMDR, Acceptable Macronutrient Distribution Range

Table e-10. Comparison of Macronutrient Intake expressed as g/day and compared to Daily Reference Intakes (DRI) [6] goal

**Mean ± SEM (range)**	**Adult (n=22)**				
	**Carbohydrate (CHO)**	**Protein (PRO)**		**FAT**	
**Estimated intake of macronutrient (g/day)***	156.9 ± 20.2 (13.9 – 464.6)	54.8 ± 4.6 (5.7- 127.3)		34.01 ± 3.3 (9.4- 82.7)	
**Macronutrient intake[a] goal (g/day)**	130	Male 56	Female 46	Male 82.6 – 103.7	Female 56.5- 64.6
**Percent predicted (%)**	120.7%	111.9%		47.2%	
**T-test *p-value***	0.43[b]	0.37[c]		<0.0001[c]	

**Mean ± SEM (range)**	**Child (n=38)[d]**							
	**Carbohydrate (CHO)**	**Protein (PRO)**					**FAT**	
**Estimated intake of macronutrient (g/day)***	142.8 ± 10.2 (32.4 – 308)	47.5 ± 3.4 (10.7 – 92.0)					40.6 ± 2.6 (13.5 – 91.1)	
**Macronutrient intake goal (g/day)**	130	1-3 yrs n=6	4-8 yrs n=6	9-13 yrs n=16	14-18 yrs		1-8 years (both sexes) 51- 65.4	
					Male n=4	Female n=4	Male 84 - 106.6	Female 69.5 – 68.7
		13	19	34	52	46		
**Percent predicted (%)**	111.2%	320.0%	260%	159.6%	55.9%	124.4%	58.2%	
		186.1%						
**T-test *p-value***	0.16[b]	<0.0001[c]					<0.0001[c]	

[a] Intake goal for CHO and PRO (g/day) is based on the DRI which is sex and age based. Population reference for Fat (g/day) intake is based on the CSFII, 1998 survey, reported in the DRI [6].

[b] Comparison of individual macronutrient intake vs. standard DRI macronutrient intake goal by unpaired parametric-test: significant difference is $p<0.05$
[c] Comparison of individual macronutrient intake vs individual macronutrient intake goal by paired parametric-test
[d]Two subjects (n=2) under 1 year of age had CHO intake goal of 95 g/day, PRO intake of 11 g/day, and fat intake of 39.5g/day

Table e-11. Macronutrient Consumption in full PMD cohort (n=60) and across Kcal intake groups				
**Mean ± SEM (range)**		**CHO consumption Kcal/day**	**PRO consumption Kcal/day**	**Fat consumption Kcal/day**
**Estimated consumption of macronutrient (Kcal/day)**		591.8 ± 39.1 (120 -1,859)	200.7 ± 10.6 (22 – 509)	391.7 ± 24.1 (84 – 859)
**WHO-MOTIVATOR predicted Kcal intake (Kcal/day)**		1,125 ± 54.4		
**Percent Macronutrient consumption of MOTIVATOR Predicted Kcal intake (%)**		48.3 ± 3.8%	15.4 ± 1%	29.4 ± 2.2%
**RDA predicted macronutrient distribution goal (mean %, AMDR*)**	**Adult**	55 (45-65%)	15 (10-35%)	30 (20-35%)
	**Pediatrics**	50 (45-65%)	20 (15-30%)	30 (25-35%)
**Percent predicted (%) when compared to macronutrient RDA goal**		94.8 ± 6.3%	80.9 ± 5.1%	84.7 ± 5.6%
**Caloric intake classification (n=60)**		**Macronutrient calorie consumption when compared to RDA goal mean ± SEM Kcal, % predicted (range)**		
		**CHO**	**PRO**	**Fat**
Excess Kcal Intake (n=17)	Subjects consumed ≥100.01%Kcal	782.1 ± 92.5 Kcal<br>143.2 ± 11.1%<br>(104.4 – 254)	216.3 ± 20.1 Kcal<br>113.9 ± 10.0%<br>(65.6 – 175.8)	412.4 ± 36.2 Kcal<br>128.8 ± 9.6%<br>(63.2 – 222.3)
Sufficient Kcal Intake (n=2)	Subjects consumed 90.01- 100% Kcal	632.4 ± 58.8 Kcal<br>94.9 ± 7.8%<br>(87.2 – 102.8)	220.6 ± 5.8 Kcal<br>82.9 ± 3.1%<br>(79.8 – 86)	406.8 ± 31.5 Kcal<br>102.0 ± 9.0%<br>(93 – 111)
Low Kcal Intake (n=12)	Subjects consumed 75.01- 90% Kcal	677.9 ± 63.6 Kcal<br>81.1 ± 3.9%<br>(62.9 -104)	214.6 ± 23.6 Kcal<br>81.9 ± 10.5%<br>(42.4 – 151.3)	422.0 ± 38.6 Kcal<br>89. ± 7.2%<br>(47.8 – 137.7)
Insufficient Kcal Intake (n=29)	Subjects consumed ≤75 % Kcal	441.9 ± 38.0 Kcal<br>51.5 ± 4.1%<br>(15.8 – 110.9)	184.5 ± 15.7 Kcal<br>61.1 ± 5.1%<br>(10.9 – 125.3)	266.2 ± 20.2 Kcal<br>54.5 ± 4.7%<br>(17.2 – 105.3)

* AMDR, Acceptable Macronutrient Distribution Range

Table e-12. Macronutrient Intake (g/day) across Kcal intake groups

**Mean ± SEM (range)**		**CHO intake g/day**	**PRO intake g/day**	**Fat intake g/day**	**Fluid intake mls/day[B]**
**Estimated intake of macronutrient**		148.0 ± 9.8 (30.1- 464.7)	50.2 ± 2.7 (5 – 127)	38.2 ± 2.1 (9.4 – 91.1)	1,333 ± 99.8 (480 – 2,640)
**Macronutrient Consumption goal[A]**		130	11- 56	39.5 – 105.6	1,819 ± 63.9 (606 – 2,724)
**Percent predicted (%)**		114.7 ± 7.5%	158.9 ± 12.2%	54.2 ± 2.8%	76.9 ± 5.0%
**Caloric intake classification (n=60)**		**Macronutrient intake (g/day) when compared to macronutrient DRI goal and CSFII survey, mean ± SEM (g/day) and fluid intake (mls/day) % predicted (range)**			
		**CHO intake g/day**	**PRO intake g/day**	**Fat intake g/day**	**Fluid intake mls/day**
Excess Kcal Intake (n=17)	Subjects consumed ≥100.01%Kcal	152.4 ±17.5 g 195.5 ± 23.1% (68.3 – 464.7)	218.3 ± 25.6 g 54.1 ± 5.0% (21.2 – 92.2)	68.3 ± 4.6 g 45.8 ± 4.0% (23.7 – 91.1)	1,280 ± 175.6 mls 85.0 ± 9.1% (38.4 – 149.5)
Sufficient Kcal Intake (n=2)	Subjects consumed 90.01- 100% Kcal	158.1 ± 14.7 g 121.6 ± 11.3% (110.3 – 132.9)	55.2 ± 1.5 g 290.3 ± 7.6% (282.6 – 297.9)	45.2 ± 3.5 g 69.1 ± 5.4% (63.8 – 74.5)	1,200 ± 120 mls 84.1 ± 11.2% (73 – 95.3)
Low Kcal Intake (n=12)	Subjects consumed 75.01- 90% Kcal	169.5 ± 7.8 g 130.4 ± 12.2% (82.2 -207.2)	53.6 ± 5.9 g 144.5 ± 18.3% (56.8 – 270.6)	46.9 ± 4.3 g 62.7 ± 5.6% (30.2 – 96)	1,503 ± 224.2 mls 72.6 ± 7.8% (38.5 – 123.9)
Insufficient Kcal Intake (n=29)	Subjects consumed ≤75 % Kcal	110.5 ± 9.5 g 85.6 ± 7.2% (23.2 – 202.5)	46.1 ± 3.9 g 120.9 ± 14.2% (10.2 - 411.5)	29.6 ± 2.2 g 41.3 ± 3.3% (14.6 – 84.3)	1,287 ± 162.3 70.8 ± 9.0% (27.6 – 147.6)

[A]Consumption goal for CHO and PRO (g/day) is based on the DRI which is sex and age based. Population reference consumption for Fat (g/day) is based on the CSFII, 1989 survey, reported in the DRI [6].
[B]Estimated Maintenance Fluid Needs was based on the Holliday Segar Equation, which is weight based.

Equation: (Patient weight – 20) * (weight based Daily fluid needs equation); adjusted body weight is used for those >20 kg [8].

Table e-13. Comparison of BMI and daily Kcal consumption with presence of GI symptoms (n=22 adult subjects*)

BMI Classification	Mean +/- SEM (range) Kcal/day	**Caloric Consumption [1] Number (%)**<br>GI Symptoms			
		**<800<br>Very Low Kcal Intake**	**801-1000<br>Low Kcal Intake**	**1000-1200<br>Moderate Kcal Intake**	**>1200<br>Appropriate Kcal Intake**
Underweight<br>n=3/22	1,179 ± 227.7<br>(815- 1598)	n=0	1 (33.3%)<br>Weight loss – 1/2<br>Constipation- 1/ 2	n=0	2 (66.7%)<br>Diarrhea^{B}- 1/2<br>Loss of Appetite^{B}-1/2
Appropriate<br>n=13/22	1,134 ± 145.4<br>(226– 2340)	2 (15.4%)<br>Dysphagia^{A}- 1/2<br>Constipation^{A}- 1/2<br>Nausea^{B}- 1/2<br>Reflux^{A}- 1/ 2<br>Vomiting^{B}- 1/2<br>GI Dysmotility^{A}- 1/2<br>Diarrhea^{B}-1/2<br>Weight loss^{B}- 1/2	3 (23.2%)<br>Dysphagia^{BC}- 2/3<br>Nausea^{A} 1/3<br>Constipation^{C}- 1/3<br>GI Dysmotility^{AB}- 2/3<br>Reflux^{AC}- 2/3<br>Vomiting^{A} 1/3<br>Weight loss^{AB}- 2/3	4 (30.8%)<br>Dysphagia^{AB}- 1/4<br>Constipation^{AB} – 2/4<br>Diarrhea^{AB}- 2/4<br>Nausea^{AB}-2/2<br>Reflux^{B}- 1/4<br>GI Dysmotility^{AB}- 2/4<br>Abdominal Pain^{B}- ¼<br>Poor weight gain^{C}-1/4<br>Weight loss^{D}- 1/4	4 (30.8%)<br>Dysphagia^{ABD}- 3/4<br>Constipation^{ABD}- 3/4<br>Failure to Thrive^{ACD}- 3/4<br>Poor weight gain^{A}- 1/4
Overweight/ Obese<br>n=6/22	1,143 ± 215.5<br>(415 – 1700)	1 (17%)<br>Weight loss- 1/1<br>Dysphagia- 1/1<br>Constipation- 1/1	2(33%)<br>Dysphagia^{A}- 1/2<br>GI Dysmotility^{A}- 1/2<br>Constipation^{A}- 1/2<br>Vomiting^{A}- 1/1	n=0	3 (42.9%)<br>Weight loss- 3/3<br>Dysphagia- 3/3<br>GI Dysmotility^{B}- 1/3<br>Constipation^{C}- 1/3<br>Nausea^{C}- 1/3<br>Reflux-3/3<br>Vomiting^{C}- 1/3<br>Diarrhea^{BC}-2/3<br>Abdominal pain^{BC}-2/3<br>Loss of appetite^{B}- 1/3
Total n (%)		3 (13.6%)	6 (27.3%)	4 (18.2%)	9 (40.9%)

*Mean weight loss for the adult cohort was -2.4 ± 0.8 kg (0.5 – 7.7), n=9/22 (40.9%).

[A/B/C/D] Individual subjects within BMI classification and Kcal classification reporting multiple GI symptoms

Table e-14. Characteristics of Weight Loss in Adult and Pediatric PMD subjects (n=16/60)		
**Mean ± SEM (Range)**	**Cohort (n=16)** Mean Interim Period of 8.8 ± 1.5 months	
**Mean Weight Loss (kg)**	-2.4 ± 0.5 kg (-7.7- -0.2)	
	**Adult (n=9)** Mean Interim Period 6.4 ± 1.3 months	**Pediatric (n=7)** Mean Interim Period 11.9 ± 2.8 months
	-2.8 ± 0.8 kg (-7.7 - -0.5)	-1.8 ± 0.5 kg (-3.4 - -0.2)
	**Pediatric Weight for age z-score (n=7) (mean ± SD) (median)**	
	Prior clinical assessment	-2.7 ± 4.1 (-1.2)
	RDN baseline assessment	-4.0 ± 5.0 (-1.2)
	Change in weight z-score	-1.3 ± 0.9
**Mean % Weight Loss (%)**	**Cohort (n=16)** Mean Interim Period of 8.8 ± 1.5 months	
	4.8 ± 0.8 % (-10.5 - -0.8)	
	**Adult (n=9)** Mean Interim Period 6.4 ± 1.3 months	**Pediatric (n=7)** Mean Interim Period 11.9 ± 2.8 months
	-4.3 ± 1.2% (-10.5 - -0.8)	-5.4 ± 1.1% (-9.0 - -1.0)
**BMI Classification of Subjects with Weight Loss**		
Underweight	5/16 (31.3%)	
Appropriate	6 /16 (37.5%)	
Overweight/Obese	5/16 (31.3%)	

Table e-15. Subjects with malnutrition (n=16) or reported weight loss (n=16) who reported relevant GI symptom				
	**Subjects with malnutrition (n=16)**	**Adult subjects (n=2/16)**	**Child subjects (n=14/16)**	**Subjects with Weight Loss (n=16)**
**Dysphagia**	11 (68.8%)	1 (50%)	10 (71.4%)	9 (56.3%)
**Constipation**	8 (50%)	1 (50%)	7 (50%)	6 (37.5%)
**Nausea**	2 (12.5%)	1 (50%)	1 (7.1%)	3 (18.8%)
**Reflux**	2 (12.5%)	0 (0.0%)	2 (14.3%)	5 (31.3%)
**Vomiting**	6 (37.5%)	1 (50%)	5 (35.7%)	5 (31.3%)
**GI dysmotility**	5 (31.3%)	0 (0.0%)	5 (35.7%)	5 (31.3%)
**Abdominal pain**	1 (6.3%)	0 (0.0%)	1 (7.1%)	4 (25.0%)
**Diarrhea**	4 (25%)	1 (50%)	3 (21.4%)	5 (31.3%)
**Loss of appetite**	4 (25%)	0 (0.0%)	4 (28.6%)	4 (25.0%)
**Difficulty feeding**	4 (25%)	0 (0%)	4 (28.6%)	1 (6.3%)
**Failure to thrive**	6 (37.5%)	0 (0%)	6 (42.9%)	3 (18.8%)

Table e-16. Malnutrition Classification in Adult and Pediatric PMD subjects

Malnutrition Guidelines Number, %	Adult (n=22)			Pediatric (n=38)		
**American ASPEN Guidelines[1]**	2 (9.1%)			14 (36.8%)		
	BMI Classification, n (%)					
	Underweight	Appropriate	Overweight/ Obese	Underweight	Appropriate	Overweight/ Obese
	0 (0%)	1 (50%)	1 (50%)	10 (71.4%)	2 (14.3%)	2 (13.3%)
**European GLIM Guidelines[9]*[a]**	2 (9.1%)			-		
	BMI Classification, n (%)					
	Underweight	Appropriate	Overweight/ Obese			
	0 (0%)	1 (50%)	1 (50%)			

Pediatric Malnutrition Severity Classification^

Levels of Malnutrition Number, %	Pediatric Subjects (n=14)
**Mild**	1 (7.1%)
**Moderate**	7 (50%)
**Severe**	6 (42.9%)

Primary Mitochondrial Disease Genetic Etiologies

Genetic Etiologies, n	**Adult Malnutrition** GLIM and ASPEN (n=2)	**Pediatric Malnutrition** ASPEN (n=14)	
**mtDNA**	MT-TK (n=1)<br>MT-TL1 (n=1)	Single, large mtDNA deletion (n=3)<br>MT-TK (n=1)<br>MT-TL1 (n=1)	MT-ND3 (n= 1)<br>MT-ND5 (n=1)<br>ATP6 (n=1)
**Nuclear**		SURF1 (n=2)<br>WARS (n=1)	ADCK3 (n=3)

[a]Malnutrition classification following GLIM (Global Leadership Initiative on Malnutrition) guidelines were only applied to the adult cohort (n =23) [9].

^Levels of Severity were only applied to the pediatric cohort following the pediatric ASPEN malnutrition criteria [2].

Table e-17. Adult Malnutrition

Adult Cohort (1-22)	Malnutrition<br>ASPEN (A) and/or GLIM(G) Criteria Met	BMI[a]	Malnutrition Classification and Criteria					Reported GI Symptoms			
			ASPEN[b]		GLIM[d]						
					Physiological Criteria		Etiological Criteria				
			Inadequate Food intake (≤ 75% REE)	Weight Loss[c]	Low BMI[e]	Weight Loss[f]	Reduced Food Intake (≤ 75% REE)	Dysphagia	GI Dysmotility	Vomiting	Reflux
Sub. 1	-	A						+			
Sub. 2	-	A						+	+		
Sub. 3	-	U									
Sub. 4	-	A	+				+	+	+		+
Sub. 5	-	U	+	+	+	+	+				
Sub. 6	-	A		+		+			+	+	+
Sub. 7	-	A			+			+			
Sub. 8	+A/G	A	+	+		+	+			+	
Sub. 9	-	A									
Sub. 10	-	O	+				+	+	+	+	
Sub. 11	-	O		+		+		+			+
Sub. 12	-	O		+		+		+	+		+
Sub. 13	-	O	+				+				
Sub. 14	-	A						+	+		+
Sub. 15	-	U			+						
Sub. 16	-	A	+				+		+		
Sub. 17	-	A	+	+		+	+	+	+		
Sub. 18	+A/G	O	+	+		+	+	+			
Sub. 19	-	A	+				+	+			
Sub. 20	-	A	+	+		+	+				
Sub. 21	-	A	+				+	+			+
Sub. 22	-	O		+		+		+		+	+

[a]BMI Classifications; U= Underweight, A= Appropriate, O= Overweight/Obese
[b]ASPEN Malnutrition requires both weight loss and inadequate food criteria to be met
[c]ASPEN criteria requires a weight loss of 5% in 1 month, 7.5% in 3 months, 10% over 6 months or 20% in 12 months
[d]GLIM Malnutrition requires both a physiological criterion (at least 1) and an etiological criterion to be met
[e]GLIM Low BMI index <20 if < 70 years
[f] GLIM weight loss of >5% within past 6 months, or >10% beyond 6 months

Table e-18. Pediatric Malnutrition

Pediatric Cohort ()	Malnutrition	BMI[a]	ASPEN Malnutrition Classification and Criteria		Reported GI Symptoms			
			BMI Z-score[b]	Reduced %Predicted Kcal Intake	Dysphagia	GI Dysmotility	Vomiting	Reflux
Sub. 1	+	U	S		+			
Sub. 2	-	O	A				+	
Sub. 3	-	A	A					
Sub. 4	-	A	A					+
Sub. 5	+	U	Mo		+		+	
Sub. 6	+	U	Mo		+		+	
Sub. 7	-	U	A					
Sub. 8	-	A	A					
Sub. 9	-	A	A					+
Sub. 10	-	A	A		+			
Sub. 11	-	O	A					
Sub. 12	-	A	A					
Sub. 13	-	U	A		+			+
Sub. 14	-	A	A					
Sub. 15	-	A	A		+	+	+	
Sub. 16	+	U	Mo					
Sub. 17	-	A	A					
Sub. 18	-	A	A					
Sub. 19	+	U	Mo		+			
Sub. 20	-	A	A					
Sub. 21	-	A	A		+	+	+	
Sub. 22	-	O	A				+	+
Sub. 23	-	A	A				+	+
Sub. 24	-	A	A					
Sub. 25	-	A	M					
Sub. 26	-	O	A					
Sub. 27	-	A	A					
Sub. 28	+	U	Mo		+	+		
Sub. 29	+	A	S	≤ 25 %	+		+	+
Sub. 30	-	A	A		+			
Sub. 31	+	U	S	≤ 25 %	+	+		
Sub. 32	+	O	Mo	(26-50%)				
Sub. 33	+	U	S		+			
Sub. 34	+	U	S	≤ 25 %	+		+	+
Sub. 35	-	A	A					
Sub. 36	+	O	Mo	(26-50%)		+		
Sub. 37	+	A	M	(51-75%)		+	+	
Sub. 38	+	U	S		+	+		

*Both BMI z-score and percent predicted Kcal intake (51-75%) criteria must be met for mild malnutrition classification per APSEN criteria
a BMI Classifications; U = Underweight, A = Appropriate, O = Overweight/Obese
b BMI Z-score Classification: A = appropriate (≥-1), M = Mild* (-1.0 to -1.99), Mo = Moderate (- 2.0 to -2.99), S = Severe (≤ -3)

Table e-19. Fluid Intake [8] based on the Recommended Daily Allowance [6]		
**Mean ± SEM<br>mls/day, range**	**Adult<br>(n=22)**	**Pediatric<br>(n=38)**
**Predicted Fluid Needs**	2,264 ± 51.3<br>(1,802 – 2,724)	1,562 ± 67.4<br>(606 – 2418)
**Estimated Daily fluid Intake**	1,704 ± 199.6<br>(n=16)	1,128 ± 91.0<br>(n=29)
**Percent predicted fluid intake (%)**	76.1%<br>(n=16)	77.3%<br>(n=29)
**T-test *p-value****	0.018	0.0003

Predicted needs (Holliday Segar Equation) vs Estimated RDN daily intake comparison by t test: significant difference (p<0.05)
*ns: no significant difference in comparison by t-test (p>0.05)
Abbreviation(s): mls - milliliters

Table e-20. Objective Assessments	
**Hand-held dynamometry muscle strength measurements**<br>Mean z-scores ± SD (95% CI)	
Elbow Flexion (n=33)	-2.4 ± 2.3 (-3.3 — -1.7)
Wrist Extension (n=32)	-3.8 ± 1.9 (-4.5 — -3.1)
Hip Flexion (n=30)	-2.2 ± 2.5 (-3.1 — -1.3)
Ankle Dorsiflexion (n=33)	-2.1 ± 2.7 (-3.1 — -1.2)
Gross Grasp (n=25)	-1.8 ± 1.3 (-2.4 — -1.3)
3-Point Pinch (n=16)	-2.9 ± 1.1 (-3.5 — -2.3)
Exercise Intolerance Assessments	
**30s STS Test**<br>Mean z-score ± SD (95% CI)<br>(n=23)	-2.01 ± 0.84<br>(-2.4 — -1.7)
**6 Minute Walk Test Total Distance**<br>Mean ± SEM (meters)<br>(n=26)	425.6 ± 20.1
**6 Minute Walk Test Total Distance**<br>Mean z-score ± SD (95% CI)<br>(n=26)	-2.9 ± 1.4<br>(-3.5 — -2.4)
**Dynamometry Repetitions on Dominant Side**<br>mean z-scores ± SD	
Elbow Flexion – 1st Repetition z-score (n=13)	-2.0 ± 1.2
Elbow Flexion – 6th Repetition z-score (n=13)	-2.9 ± 1.1
Negative Percent Decrement (%) (n=13)<br>(mean ± SEM)	-15.3 ± 2.9

Table e-21. Quality of Life, Fatigue and Performance Survey Results		
**Survey**	**Scores**	**Scoring Details**
**Pediatric Quality of Life Inventory** [10] **(PedsQL)**[A]	Mean z-score ± SD	Scores are standardized by specific age groups, 2-4, 8-12, 13- 17 and 18+ years, as well as proxy vs self-completed.
Total Score (n=37)	-1.9 ± 1.5	
Physical Health Summary Score	-2.1 ± 2.1	
Psychosocial Health Summary Score	-1.4 ± 1.2	
School/Work Functioning Score	-1.0 ± 0.83	
**Modified Fatigue Impact Scale [11] (MFIS)**[B]	Mean score ± SEM (range)	Cut off score for fatigue is 38.
Total Score (n=33)	40.1 ± 2.8<br>(9 – 74)	
**Karnofsky/Lansky Performance Scale [12] (K/L Scale)**[C]	Mean Score ± SEM (range)	Scale has a score range of 10-100.<br><br>Score 10- 40: Moderate to Severe Restrictions<br>Score 50-80: Effortful activity/Restricted in strenuous play
Total Score (n=37)	71.6 ± 3.2<br>(30 – 100)	
**Sec. IV Newcastle Mitochondrial Disease Adult (SF12v2)/Pediatric Scale**[D] **Section 4/ SF-12 [13] (NMDAS/NMDPS)**	Mean Score ± SD	
SF12v2 Transformed Physical Score (n=7)	28.6 ± 8.6	Score of 50 represents the function of the general population (SD of 10). Scores <50 reflect worse function than general population.
SF12v2 Transformed Mental Score (n=7)	50.0 ± 9.8	

NPMDS Sec IV Final Score (n=7)	11.3 ± 4.2	Scale has a score range from between 0-25, ranging from very good (0) to very poor quality of life (25).
**Pediatric Evaluation of Disability Inventory Computer Adaptive Test (PEDI-CAT)**[E]	Mean Z-Score ± SD (range)	
Daily Activities (n=11)	-1.7 ± 2.1 (-5.4 – 0.7)	Score of 0 represents the function of the general populations (SD of 2). Z-scores < -2 reflect worse performance/skill in the domain than general population.
Mobility (n=11)	-3.7 ± 3.5 (-9.0 – 0.1)	
Social/Cognitive (n=11)	-1.6 ± 1.9 (-6.2 – 0.3)	
Responsibility (n=6)	-1.7 ± 1.7 (-4.6 - -0.3)	

[A] Pediatric Quality of Life Inventory is a 23-item questionnaire that evaluates the health-related quality of life, consisting of 4 functioning scales that then equate to 3 summary scores. The Physical Health Summary score consist of 8 items, the Psychosocial Health Summary Score consists of a mix of 15 items and the School/Work Functioning score consist of 5 items.

[B] The Modified Fatigue Impact Scale measures the impact of fatigue on a person's activities. The higher the total score, the greater the impact fatigue has on the person.

[C] The Karnofsky/Lansky Performance Scale allows for the functional status of a subject to be determined through a series of questions in which the total score is placed on a scaling system from 10-100, with functional descriptions in 10-point intervals. The higher scores are associated with increased functional status.

[D]NMDAS/NMDPS section 4 and SF 12v2 are sections that specifically evaluates quality of life and is filled out by only the patient/caregiver only. The NMDAS SF12v2 have transformed physical and mental scores while the NPMDS section 4 has a final tabulated score.

[E] The PEDI-CAT is a 4 sub-section test that evaluates the overall functionality, performance and skills of a subject. A z-score of 0 represents the function of the general population (SD of 2). A z-score below -2 reflects worsening performance/skill in the domain than the general population. The four domains of the PEDI-CAT are daily activities, mobility, social/cognitive and responsibility.

Table e-22. Correlations of objective assessments and surveys to macronutrient consumption (g/day) for all subjects (n=60)

% predicted values	**Dominant Elbow Flexion (n=33)**		**Dominant Wrist Extension (n=32)**		**Dominant Hip Flexion (n=30)**		**Dominant Ankle Dorsiflexion (n=33)**		**6MWT Total Distance Walked (n=26)**		**Exercise Intolerance - 30s STS (n=23)**	
	r	*p-value*	r	*p-value*	r	*p-value*	r	*p-value*	r	*p-value*	r	*p-value*
**CHO g/day**	-0.20	0.26	0.03	0.87	-0.22	0.24	-0.002	0.99	-0.20	0.34	-0.11	0.60
**PRO g/day**	0.10	0.55	0.28	0.13	0.14	0.47	0.32	0.07	0.03	0.89	-0.01	0.95
**Fat g/day**	-0.09	0.60	0.16	0.39	-0.17	0.37	0.29	0.11	-0.01	0.96	0.04	0.87

% predicted values	**Muscle Fatigue - Elbow Flexion (z-score) (n=13)**		**Elbow Flexion (negative % decrement) (n=13)**		**PedsQL Total Function Score (n=37)**		**PedsQL Physical Function Score (n=37)**		**PedsQL Psychosocial Function Score (n=37)**		**PedsQL School/work Function Score (n=26)**	
	r	*p-value*	r	*p-value*	r	*p-value*	r	*p-value*	r	*p-value*	r	*p-value*
**CHO g/day**	-0.51	0.24	-0.14	0.66	0.14	0.42	0.17	0.31	0.05	0.75	0.17	0.41
**PRO g/day**	-0.61	0.03	-0.70	0.009	0.63	<0.0001	0.56	0.0003	0.56	0.0003	0.27	0.18
**Fat g/day**	-0.75	0.004	-0.39	0.18	0.34	0.04	0.35	0.03	0.24	0.16	0.24	0.24

% predicted values	**MFIS Total Score (n=33)**		**Karnofsky/Lansky Performance Score (n=37)**		**PEDI-CAT Daily Activities z-score (n=11)**		**PEDI-CAT Mobility z-score (n=11)**		**PEDI-CAT Social/Cognitive z-score (n=11)**	
	r	*p-value*	r	*p-value*	r	*p-value*	r	*p-value*	r	*p-value*
**CHO g/day**	-0.14	0.43	-0.01	0.73	0.15	0.67	0.10	0.78	0.14	0.69
**PRO g/day**	-0.27	0.12	0.03	0.88	0.50	0.12	0.74	0.01	0.74	0.01
**Fat g/day**	-0.10	0.59	0.08	0.66	0.14	0.69	0.46	0.15	0.40	0.23

Table e-23. Correlations of objective assessments and surveys to macronutrient consumption (% Kcal) for all subjects (n=60)

% predicted values	Dominant Elbow Flexion (n=33)		Dominant Wrist Extension (n=32)		Dominant Hip Flexion (n=30)		Dominant Ankle Dorsiflexion (n=33)		6MWT total distance walked (n=26)		Exercise Intolerance - 30s STS (n=23)	
	r	*p-value*	r	*p-value*	r	*p-value*	r	*p-value*	r	*p-value*	r	*p-value*
**CHO Kcal**	-0.20	0.27	-0.03	0.88	-0.21	0.30	-0.11	0.61	-0.21	0.30	-0.11	0.61
**PRO Kcal**	0.13	0.44	-0.16	0.37	-0.20	0.32	0.05	0.83	-0.20	0.32	0.05	0.83
**Fat Kcal**	-0.20	0.27	0.004	0.98	-0.07	0.73	0.10	0.65	-0.07	0.73	0.10	0.65
% predicted values	**Muscle Fatigue-Elbow Flexion (z-score) (n=13)**		**Elbow Flexion (negative % decrement) (n=13)**		**PedsQL Total z-score (n=37)**		**PedsQL Physical Function z-score (n=37)**		**PedsQL Psychosocial Function z-score(n=37)**		**PedsQL School/work Function z-score (n=26)**	
	r	*p-value*	r	*p-value*	r	*p-value*	r	*p-value*	r	*p-value*	r	*p-value*
**CHO Kcal**	-0.48	0.10	-0.06	0.85	0.17	0.33	0.15	0.38	0.19	0.25	0.24	0.25
**PRO Kcal**	-0.54	0.06	-0.82	0.001	-0.04	0.83	-0.18	0.29	0.28	0.09	0.39	0.0496
**Fat Kcal**	-0.67	0.02	-0.14	0.66	0.29	0.09	0.28	0.10	0.29	0.08	0.29	0.15
% predicted values	**MFIS Total Score (n=33)**		**Karnofsky/Lansky Performance Score (n=37)**		**PEDI-CAT Daily Activities z-score (n=11)**		**PEDI-CAT Mobility z-score (n=11)**		**PEDI-CAT Social/Cognitive z-score (n=11)**			
	r	*p-value*	r	*p-value*	r	*p-value*	r	*p-value*	r	*p-value*		
**CHO Kcal**	-0.14	0.44	-0.24	0.16	0.19	0.58	0.49	0.13	0.74	0.01		
**PRO Kcal**	0.15	0.41	-0.26	0.12	0.45	0.17	0.56	0.08	0.67	0.03		
**Fat Kcal**	-0.04	0.85	-0.11	0.53	0.06	0.88	0.44	0.18	0.51	0.11		

Table e-24. Correlations between surveys and daily Kcal intake	
	%Predicted daily Kcal intake
**PedsQL Total z-score**	
Number (n)	37
r	0.19
p-value	0.25
**PedsQL Physical Function z-score**	
Number (n)	37
r	0.15
p-value	0.39
**PedsQL Psychosocial Function z-score**	
Number (n)	37
r	0.28
p-value	0.10
**PedsQL School/Work Function z-score**	
Number (n)	26
r	0.35
p-value	0.08
**MFIS Total Score**	
Number (n)	33
R	-0.06
p-value	0.74
**Karnofsky/Lansky Performance Score**	
Number (n)	37
r	-0.19
p-value	0.26
**PEDI-CAT Daily Activities z-score**	
Number (n)	11

r	0.20
p-value	0.56
**PEDI-CAT Mobility z-score**	
Number (n)	11
r	0.60
p-value	0.06
**PEDI-CAT Social/Cognitive z-score**	
Number (n)	11
r	0.78
p-value	0.006

Table e-25. Correlations of objective assessments and surveys to macronutrient consumption (g/day) for subjects with inadequate Kcal intake (≤75% predicted) (n=29)

% predicted values	Dominant Elbow Flexion (n=20)		Dominant Wrist Extension (n=18)		Dominant Hip Flexion (n=19)		Dominant Ankle Dorsiflexion (n=21)		6MWT Total Distance Walked (n=20)		Exercise Intolerance - 30s STS (n=16)	
	r	*p-value*	r	*p-value*	r	*p-value*	r	*p-value*	r	*p-value*	r	*p-value*
**CHO g/day**	-0.23	0.34	0.44	0.07	-0.29	0.24	-0.12	0.60	-0.32	0.17	-0.32	0.23
**PRO g/day**	0.03	0.90	0.31	0.21	0.22	0.37	0.32	0.15	0.13	0.58	0.12	0.66
**Fat g/day**	-0.12	0.61	0.41	0.09	-0.13	0.59	0.44	0.047	0.10	0.67	0.10	0.70

% predicted values	PedsQL Total z-score (n=19)		PedsQL Physical Function z-score (n=19)		PedsQL Psychosocial Function z-score (n=19)		PedsQL School/work Function z-score (n=14)		MFIS Total Score (n=16)		Karnofsky/Lansky Performance Score (n=18)	
	r	*p-value*	r	*p-value*	r	*p-value*	r	*p-value*	r	*p-value*	r	*p-value*
**CHO g/day**	0.10	0.70	0.16	0.51	-0.40	0.13	0.04	0.89	-0.06	0.84	-0.42	0.08
**PRO g/day**	0.54	0.02	0.41	0.09	0.47	0.04	0.33	0.25	-0.22	0.41	-0.04	0.88
**Fat g/day**	0.38	0.10	0.35	0.14	0.02	0.95	0.28	0.33	-0.06	0.84	0.07	0.77

Table e-26: Correlations of objective assessments and surveys to macronutrient consumption (Kcal) for subjects with inadequate Kcal intake (≤75% predicted) (n=29)

% predicted values	Dominant Elbow Flexion (n=20)		Dominant Wrist Extension (n=18)		Dominant Hip Flexion (n=19)		Dominant Ankle Dorsiflexion (n=21)		6MWT Total Distance Walked (n=20)		Exercise Intolerance - 30s STS (n=16)	
	r	*p-value*	r	*p-value*	r	*p-value*	r	*p-value*	r	*p-value*	r	*p-value*
**CHO Kcal**	-0.23	0.34	0.35	0.16	-0.24	0.31	-0.07	0.76	-0.21	0.38	-0.13	0.63
**PRO Kcal**	0.11	0.66	-0.02	0.95	0.53	0.02	0.05	0.83	0.09	0.71	0.00	1.0
**Fat Kcal**	-0.27	0.25	0.16	0.53	-0.24	0.33	0.27	0.24	0.11	0.65	0.34	0.20

% predicted values	PedsQL Total score (n=19)		PedsQL Physical Function z-score (n=19)		PedsQL Psychosocial Function z-score (n=19)		PedsQL School/work Function z-score (n=14)		MFIS Total Score (n=16)		Karnofsky/Lansky Performance Score (n=18)	
	r	*p-value*	r	*p-value*	r	*p-value*	r	*p-value*	r	*p-value*	r	*p-value*
**CHO Kcal**	0.11	0.67	0.44	0.09	0.17	0.49	0.07	0.81	0.04	0.88	-0.24	0.34
**PRO Kcal**	0.14	0.56	-0.09	0.75	-0.11	0.65	0.32	0.27	-0.03	0.92	-0.12	0.65
**Fat Kcal**	0.29	0.23	0.24	0.36	0.34	0.16	0.29	0.32	0.09	0.73	-0.01	0.97

References

1. The ASPEN Adult nutrition support core curriculum. Silver Spring, MD American Society for Parenteral and Enteral Nutrition; 2007.
2. The ASPEN Pediatric Nutrition Support Core Curriculum,. 2nd ed2015.
3. Energy and protein requirements. Report of a joint FAO/WHO/UNU Expert Consultation. World Health Organization technical report series. 1985;724:1-206.
4. Harris JA, Benedict FG. A Biometric Study of Human Basal Metabolism. Proc Natl Acad Sci U S A. 1918;4(12):370-3.
5. Mifflin MD, St Jeor ST, Hill LA, Scott BJ, Daugherty SA, Koh YO. A new predictive equation for resting energy expenditure in healthy individuals. Am J Clin Nutr. 1990;51(2):241-7.
6. Council NR. Recommended Dietary Allowances: 10th Edition. Washington, DC: The National Academies Press; 1989. 302 p.
7. Zweers HEE, Bordier V, In 't Hulst J, Janssen MCH, Wanten GJA, Leij-Halfwerk S. Association of Body Composition, Physical Functioning, and Protein Intake in Adult Patients With Mitochondrial Diseases. JPEN J Parenter Enteral Nutr. 2020.
8. Holliday MA, Segar WE. The maintenance need for water in parenteral fluid therapy. Pediatrics. 1957;19(5):823-32.
9. Cederholm T, Jensen GL, Correia M, Gonzalez MC, Fukushima R, Higashiguchi T, et al. GLIM criteria for the diagnosis of malnutrition - A consensus report from the global clinical nutrition community. Clinical nutrition (Edinburgh, Scotland). 2019;38(1):1-9.
10. Varni JW, Seid M, Kurtin PS. PedsQL 4.0: reliability and validity of the Pediatric Quality of Life Inventory version 4.0 generic core scales in healthy and patient populations. Medical care. 2001;39(8):800-12.
11. Larson RD. Psychometric properties of the modified fatigue impact scale. International journal of MS care. 2013;15(1):15-20.
12. McKay MJ, Baldwin JN, Ferreira P, Simic M, Vanicek N, Hiller CE, et al. 1000 Norms Project: protocol of a cross-sectional study cataloging human variation. Physiotherapy. 2016;102(1):50-6.
13. Schaefer AM, Phoenix C, Elson JL, McFarland R, Chinnery PF, Turnbull DM. Mitochondrial disease in adults: a scale to monitor progression and treatment. Neurology. 2006;66(12):1932-4.
